# Direct Observation of Ni Nanoparticle Growth in Carbon-Supported Nickel under Carbon Dioxide Hydrogenation Atmosphere

**DOI:** 10.1021/acsnano.3c03721

**Published:** 2023-07-28

**Authors:** Nienke L. Visser, Savannah J. Turner, Joseph A. Stewart, Bart D. Vandegehuchte, Jessi E. S. van der Hoeven, Petra E. de Jongh

**Affiliations:** † Materials Chemistry and Catalysis, Debye Institute for Nanomaterials Science, Utrecht University, Universiteitsweg 99, 3584 CG Utrecht, The Netherlands; ‡ TotalEnergies OneTech Belgium, B-7181 Seneffe, Belgium

**Keywords:** *in situ* transmission electron microscopy, nickel, CO_2_ hydrogenation, catalyst stability, nanoparticle growth mechanisms, diffusion and coalescence, Ostwald ripening

## Abstract

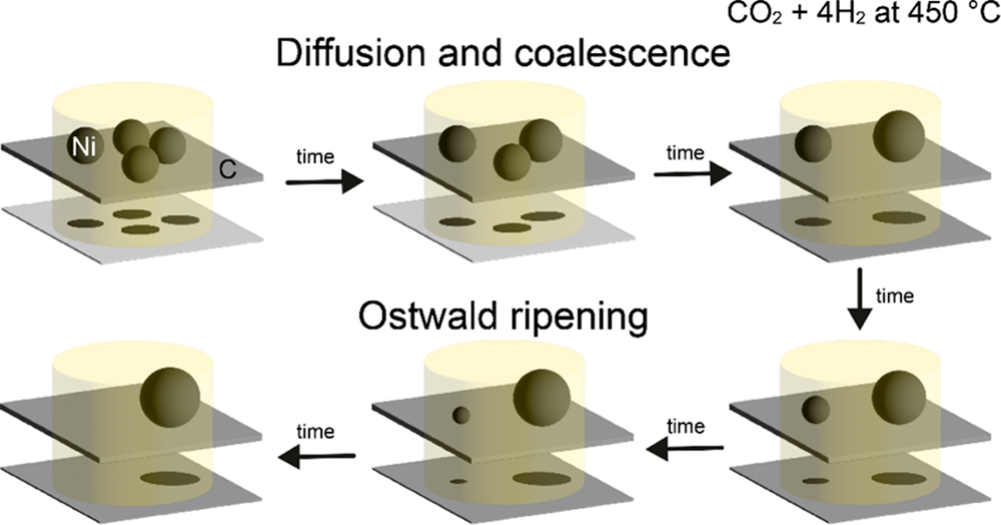

Understanding nanoparticle growth is crucial to increase the lifetime of supported metal catalysts. In this study, we employ *in situ* gas-phase transmission electron microscopy to visualize the movement and growth of ensembles of tens of nickel nanoparticles supported on carbon for CO_2_ hydrogenation at atmospheric pressure (H_2_:CO_2_ = 4:1) and relevant temperature (450 °C) in real time. We observe two modes of particle movement with an order of magnitude difference in velocity: fast, intermittent movement (*v*_max_ = 0.7 nm s^–1^) and slow, gradual movement (*v*_average_ = 0.05 nm s^–1^). We visualize the two distinct particle growth mechanisms: diffusion and coalescence, and Ostwald ripening. The diffusion and coalescence mechanism dominates at small interparticle distances, whereas Ostwald ripening is driven by differences in particle size. Strikingly, we demonstrate an interplay between the two mechanisms, where first coalescence takes place, followed by fast Ostwald ripening due to the increased difference in particle size. Our direct visualization of the complex nanoparticle growth mechanisms highlights the relevance of studying nanoparticle growth in supported nanoparticle ensembles under reaction conditions and contributes to the fundamental understanding of the stability in supported metal catalysts.

Nickel-based catalysts are often used for the hydrogenation of carbon dioxide. An example is the so-called Sabatier reaction, in which CO_2_ and renewable hydrogen are converted into synthetic natural gas (power-to-gas process).^1−3^ The catalytic performance in terms of activity is controlled by the available surface area of the metal nanoparticles.^4−6^ The exposure of nanoparticles to reaction atmospheres and elevated temperatures can result in nickel particle growth and activity loss. The thermodynamic driving force of nanoparticle growth is the minimization of surface energy, which is higher for smaller nanoparticles that contain a large fraction of uncoordinated surface atoms.^7^ Identifying the nanoscale growth mechanism during catalyzed processes is important to understand and improve catalyst stability. Metal nanoparticle growth in supported catalysts is typically attributed to two mechanisms: (i) particle diffusion and coalescence, where mobile particles randomly diffuse over the support, collide, and merge when in close proximity,^7−9^ and (ii) Ostwald ripening, where large particles grow at the expense of smaller ones via mobile metal species that travel over the support surface or via the gas phase.^10^ This process is driven by the Gibbs–Thomson effects,^11^ where small particles are more likely to release single atoms,^8^ resulting in a lower stability. Unraveling the growth mechanisms can be done indirectly, based on the catalytic activity as a function of time^12^ and/or via *ex situ* characterization by analyzing the particle size distributions before and after catalysis.^13,14^ However, if both growth mechanisms occur simultaneously,^15−17^ identification based on catalytic data or *ex situ* characterization does not suffice^18,19^ and the direct observation of nanoparticle growth under reaction conditions is required.

In catalysis, it is important to overcome the “pressure gap” and reach an understanding of nanoscale phenomena under conditions that are relevant for real applications.^20−25^ The recent advances in *in situ* transmission electron microscopy (TEM) using closed-cell nanoreactors, in which samples can be exposed to gas- or liquid-phase environments, allow the visualization of nanoparticle catalysts under 1 bar gas. This enables the analysis of changes in nanoparticle morphology, size, composition and shape of metal nanoparticles under reaction conditions, which is highly relevant to the field of catalysis in general^26,27^ and in particular to the field of nanoparticle stability.^28−31^ With this technique, both the diffusion and coalescence and the Ostwald ripening processes can be visualized^13,16,28,31^ and the main mechanism is most likely dependent on the catalyst, gas atmospheres, and conditions used.

Reproducing the same behavior of a catalyst from a reactor setup in an *in situ* TEM holder is not evident.^20,32^ The performance of a catalyst is affected by factors such as temperature, gas hourly space velocity (GHSV), grain size, and type of reactor used. Thus, differences in the flow dynamics of a gas cell compared to a catalytic reactor can affect the observed results.^33^ Moreover, the nature of supported nanoparticles with nonstructured, high-surface-area supports used for heterogeneous catalysis makes it inherently challenging to analyze these catalysts with electron microscopy and in particular during *in situ* studies. As a result, *in situ* TEM studies are often performed with unsupported nanoparticles dispersed on the chip^34,35^ or on smooth planar^36^ or nonporous spherical supports,^37,38^ while performing high-resolution analysis on only a few nanoparticles.^34,35,37,39,40^ Studies related to CO_2_ hydrogenation at atmospheric pressure have been performed using unsupported (TEM grid or window supported) nanoparticles e.g. of In_2_O_3_,^41^ Au,^42^ Ni,^35^ and AuNi,^43^ studying a few supported nanoparticles, e.g. using Rh/TiO_2_,^44^ Ni/TiO_2_^45^ for methanation or studying an industrial Cu/ZnO/Al_2_O_3_ methanol synthesis catalyst.^46^ A strong metal support interaction has been reported in a combined CO_2_ and H_2_ atmosphere for Rh/TiO_2_ methanation catalysts, limiting nanoparticle growth,^44^ whereas some growth was observed for Ni/TiO_2_ under CO_2_ hydrogenation conditions, but not studied in detail.^45^

Here, we visualize nanoparticle growth for tens of nanoparticles in a nickel on carbon catalyst under relevant reaction atmospheres and elevated temperatures using *in situ* gas-phase TEM. Previously, we showed that these nickel on carbon catalysts are active CO_2_ hydrogenation catalysts under industrially relevant pressures and temperatures.^4,47^ Over the course of 100 h on stream, the catalyst lost part of its activity, likely due to metal nanoparticle growth.^47^ The low contrast and sheetlike nature of the carbon used (graphitic nanoplatelets, GNP) make these catalysts particularly attractive for *in situ* TEM characterization. In this work, nanoparticle movement and growth of the nickel on carbon catalyst were directly visualized during exposure to CO_2_ hydrogenation conditions. We identified different types of movement and growth mechanisms, occurring simultaneously within the same sample.

## Results and Discussion

### Averaged *In Situ* Ni Particle Growth

The catalysts used for this study contained nickel metal nanoparticles on a sheet-like graphitic carbon support with a surface area of 456 m^2^ g^–1^ (graphitic nanoplatelets, GNP).^47^ The average size of the Ni nanoparticles in the fresh catalyst was 4.6 ± 1.2 nm, determined using TEM under vacuum, and the X-ray diffraction (XRD) crystallite size was 4.2 nm. Complete characterization can be found in ref (47). Before discussing the results of the *in situ* observations in detail, it is important to first study the averaged particle growth and validate the observed *in situ* TEM results, as is done in the first two sections.

Unless stated otherwise, all *in situ* TEM results were obtained at 450 °C, 1 bar, and 4:1 H_2_:CO_2_ gas with a flow rate of 0.4 sccm. This temperature was chosen as it is still in the regime that is relevant for catalysis, although on the high side, to accelerate the growth process and to allow its visualization within a limited period of electron microscopy time. We established in a catalytic reactor setup that a temperature of 450 °C was sufficient for this catalyst to show activity for CO_2_ hydrogenation at 1 bar, with 23% CO_2_ conversion, under high GHSV (1.3 × 10^6^ mL g_cat_ h^–1^), converting CO_2_ into CO and CH_4_. Only a small fraction (<1%) of the CH_4_ formed was caused by support methanation, which was checked by a second test, where the CO_2_ was replaced with N_2_, hence in the presence of only H_2_ as reaction gas (Table S1). We note that this GHSV is still orders of magnitude lower than that in the *in situ* TEM holder. However, a smaller amount of catalyst in the catalytic reactor is not feasible, due to sensitivity limitations of the analytics.

**Figure 1:**
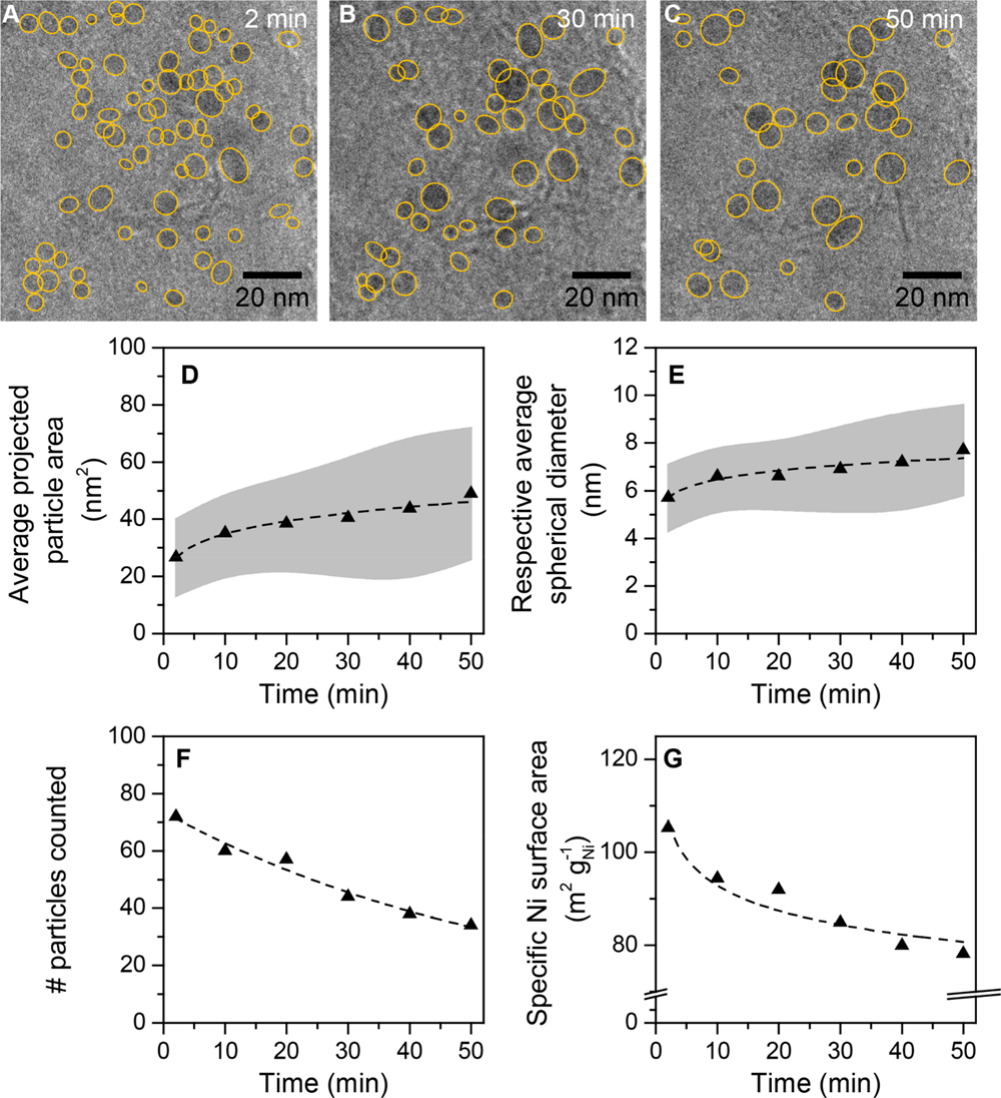
*In situ* analysis of the average nanoparticle growth: Transmission electron microscopy images of Ni/GNP acquired after (A) 2, (B) 30. and (C) 50 min. The perimeter of all observed particles is highlighted in yellow. (D) Average projected particle area and (E) average particle diameter calculated from the projected particle areas assuming a spherical shape, as a function of time. In gray is shown the standard deviation of the measurements. (F) Total number of particles in the field of view and (G) specific metal surface area as a function of time. Dashed lines are added as guides to the eye. During the experiment, the imaged areas were exposed to an electron dose of 8 e^–^ s^–1^ A^–2^ every 2 min for ∼10 s.

Figure 1A–C displays TEM images from the time-lapsed series of a selected region of 108 × 109 nm in which the perimeters of all observed nanoparticles are highlighted. The selected TEM images taken at *t* = 2 min (Figure 1A), *t* = 30 min (Figure 1B), and *t* = 50 min (Figure 1C) demonstrate that over time particle growth occurred and the number of Ni particles in the field of view decreased. TEM images of the catalyst directly after reduction and at certain stages before the start of the actual *in situ* CO_2_ hydrogenation experiment (see Methods) can be found in Figure S1. As the field of view in Figure 1A–C contains a large number of individual Ni particles, it was used for the analysis of the overall evolution in Ni nanoparticle size, expressed as the average particle area (Figure 1D) and the average diameter assuming a spherical shape (Figure 1E). The particle diameter increased from *d* = 5.7 ± 1.4 nm to *d* = 7.7 ± 1.9 nm (Figure 1E), and the number of particles decreased from 72 at *t* = 2 min to 34 at *t* = 50 min (Figure 1F). Using these data, we verified that the total weight of nickel in the field of the view remained roughly constant: 7.2 × 10^–17^ and 8.4 × 10^–17^ g nickel at *t* = 0 and *t* = 50 min, respectively. The small increase in mass over time may indicate the presence of particles that were initially too small to analyze (*d* < 2 nm). The relevant parameter for catalysis is the Ni surface area, the evolution of which is displayed Figure 1G. To calculate the specific surface areas, the particle diameters of Figure 1E were used, assuming a spherical particle shape (see Methods for the calculation details). Figure 1G shows a decrease in specific Ni active surface area from 105 to 78 m^2^ g_Ni_^–1^ in the first 50 min under CO_2_ hydrogenation conditions. As catalytic reactions take place on the surface of the metal nanoparticles, a decrease in active metal surface area due to particle growth is directly related to catalyst activity loss over time^12,47−50^ and is hence undesirable.

### Validating the *In Situ* TEM Results

**Figure 2:**
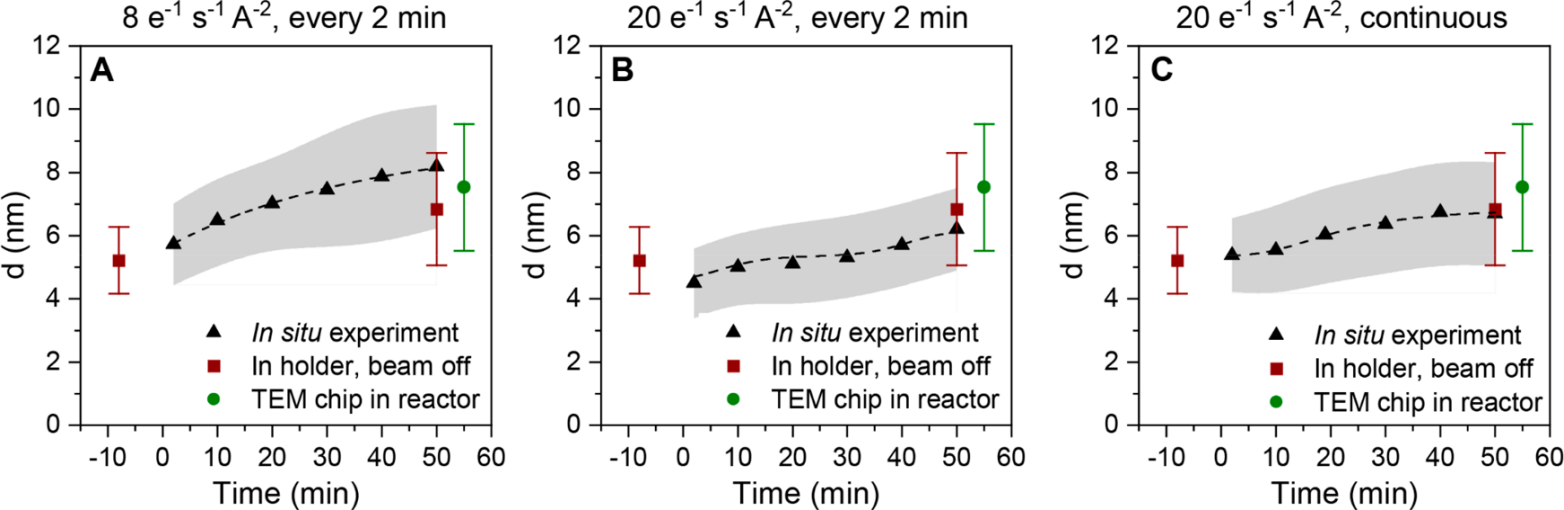
Validation of the *in situ* TEM results. Averaged particle diameters assuming spherical particles versus time during of exposure to a flow of 4:1 CO_2_:H_2_ gas at 1 bar and 450 °C and various electron beam doses: (A) 8 e^–^ s^–1^ A^–2^ every 2 min for ∼10 s (data shows a combination of two analyzed regions), (B) 20 e^–^ s^–1^ A^–2^ every 2 min for ∼10 s, and (C) 20 e^–^ s^–1^ A^–2^ continuously (data show a combination of two analyzed regions). In gray is shown the standard deviation of the measurements. The dashed lines are used as guides for the eye. To investigate beam effects, the experiments were repeated on a TEM chip in the *in situ* TEM holder without exposure to the beam (dark red squares) and on a TEM chip in a fixed bed reactor (green circles).

It is crucial to realize that, despite the observation of tens of nanoparticles, these data concern a small amount of catalyst (∼10^–15^ g). It is thus important to verify whether the observations are representative for the whole sample. Figure 1 was part of a larger field of view (380 × 380 nm), which is displayed in Figure S2 and Movie S1. A first check was performed by analyzing a second region from this experiment. The number of particles decreased from 87 at *t* = 2 min to 55 *t* = 50 min with a respective particle growth from *d* = 5.8 ± 1.2 nm to *d* = 8.5 ± 1.9 nm (shown in Figure S3), which corroborated the findings of region 1. The average particle diameter for the two measured regions increased from *d* = 5.7 ± 1.3 nm to *d* = 8.2 ± 2.0 nm (Figure 2A). Second, we found that under a pure Ar atmosphere, barely any particle growth was observed with *in situ* TEM (Figure S4), confirming that the growth observed was not only a temperature effect but also a result of exposure of the catalyst to reaction gases.

It is known that the electron beam can drastically influence the results of *in situ* experiments, for example due to radiolysis of the gas phase, the formation of (dilute) plasma, or knock-on damage.^51,52^ Therefore, the electron dose during *in situ* experiments was optimized by conducting *ex situ* particle growth studies. To achieve sufficient contrast, especially to visualize the smallest particles, an electron dose rate of up to 20 e^–^ A^–2^ s^–1^ was needed. We employed two strategies to evaluate the impact of the increased electron dose compared to 8 e^–^ A^–2^ s^–1^ used in the experiment of Figure 1. In the first control experiment, the beam was only turned on every 2 min for ∼10 s with an e^–^ dose rate of 20 e^–^ A^–2^ s^–1^ to take an image (resulting in an average dose rate below 2 e^–^ A^–2^ s^–1^, which was comparable to another *in situ* EM study on carbon materials^53^). This resulted in particle growth from *d* = 4.5 ± 1.1 nm to *d* = 6.2 ± 1.3 nm (Figure 2B). The details are shown in Movie S2 and Figures S5 and S6. In the second experiment (Movie S3) the sample was continuously exposed to the electron beam with a dose rate of 20 e^–^ A^–2^ s^–1^ and an image was taken every 2 s to visualize processes that occur on a short time scale (details shown in Figures S7 and S8). Here, particle growth from *d* = 5.4 ± 1.2 nm to *d* = 6.7 ± 1.6 nm (averaged over two measured regions) was observed (Figure 2C). The results are summarized in Figure 2 and shown in more detail in Table S2. The averaged particle sizes at the start and end of both control experiment were, within the error of a measurement, comparable to the results shown in Figure 1, and thus there was no significant effect of increasing the electron beam dose from 8 to 20 e^–^ A^–2^ s^–1^ for our catalyst system and conditions. The relatively large standard deviation was mainly caused by the polydispersity of the sample. For completeness, also the median and geometric mean of all measurements were calculated, which were similar to the number-averaged particle sizes for all experiments (Table S3). Hence, the results of the three experiments are used in this study, always clearly stating the electron dose rate used.

We further excluded significant effects of the electron beam by a direct comparison to two *ex situ* control experiments (outside the electron microscope) under similar conditions (Figure 2). First, the experiment was repeated in the TEM holder without exposure to an electron beam under the same gas atmosphere (details in Figure S9). Second, a TEM chip with a sample was put in a fixed bed reactor, where the reaction conditions from the *in situ* experiments were mimicked (details in Figure S10). The main outcomes are included in Figure 2, and a full overview is given in Table S2. For all experiments, the averaged particle diameters were, within the standard deviation, similar both at the start and at the end of the experiments compared to all *in situ* experiments. Based on these results we are confident that the growth of the Ni nanoparticles observed with *in situ* TEM was not significantly affected by the electron beam at the dose rates and conditions used.

### Particle Movement

**Figure 3:**
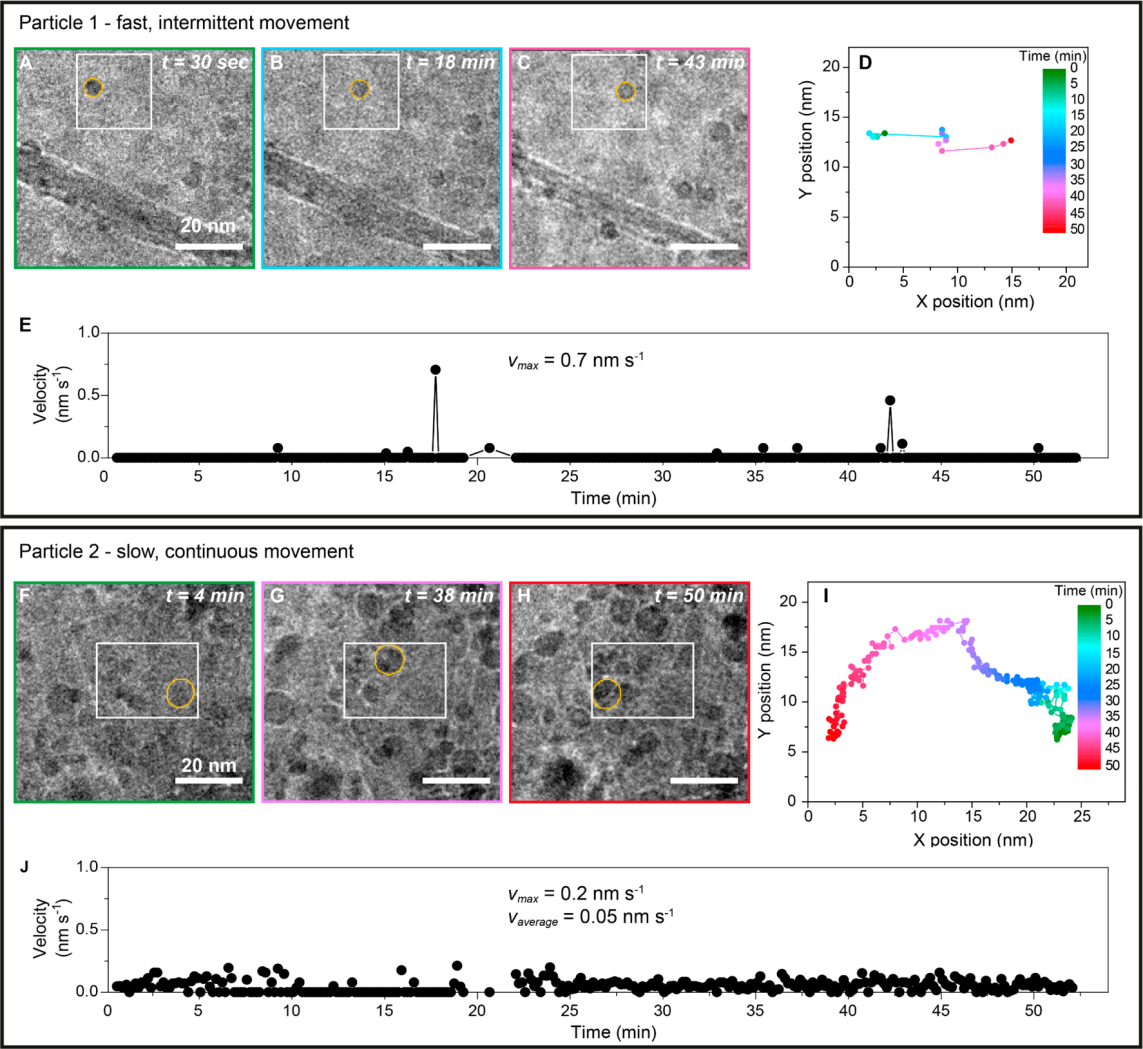
Examples of particle movement studied *in situ*. TEM images highlight particle 1 (top panel) at (A) *t* = 30 s, (B) *t* = 18 min, and (C) *t* = 43 min and particle 2 (bottom panel) at (E) *t* = 4 min, (F) *t* = 38 min, and (G) *t* = 50 min. The white boxes indicate the frame used to determine the *X* and *Y* location of the nanoparticles. (D, I) Tracks of the center of the analyzed nanoparticles in the corresponding row. (E, J) Velocity versus time of the nanoparticles in the corresponding panel. During the experiment, the imaged areas were continuously exposed to an electron dose of 20 e^–^ A^–2^ s^–1^_._.

The *in situ* TEM experiments allowed a detailed view on the how particles moved over the support. Especially in the experiment where the images were collected continuously (Movie S3), two types of movement were observed: (i) slow, continuous movement and (ii) fast, intermittent movement. Examples of both types of movement are highlighted in Figure 3. Figure 3A–C shows TEM images from the time-lapsed series highlighting a nanoparticle (particle 1) that at first is immobile, then suddenly moves over several nanometers and after that is immobile again. This phenomenon occurred twice and both times within 10 s (the time frame of analysis), which means that average velocities up to 0.7 nm s^–1^ were reached (Figure 3E).

Alternatively, more gradual movement was observed for particle 2, highlighted in the TEM images in Figure 3F–H, which moved over the course of the entire experiment (Figure 3I), covering a distance of ca. 147 nm during 50 min (Figure 3J). Its velocity reached a maximum of 0.2 nm s^–1^, and the average velocity was 0.05 nm s^–1^. Single nanoparticles were not limited to only one type of movement. Figure S11 shows an example where both fast, intermittent (*v* = 0.6 nm s^–1^) and slow, random movement at a lower velocity (*v* < 0.2 nm s^–1^) were observed over the course of the entire experiment. Interestingly, for all experiments we found that particles located at the edges of the graphene sheets did not move significantly (Movie S2), which could indicate that they are stabilized by support defects. Hence from the data in Figure 3, we show two types of movement: an intermittent movement with velocities up to 0.7 nm s^–1^ and a slower gradual movement where the average velocity was an order in magnitude lower (on average 0.05 nm s^–1^).

**Figure 4:**
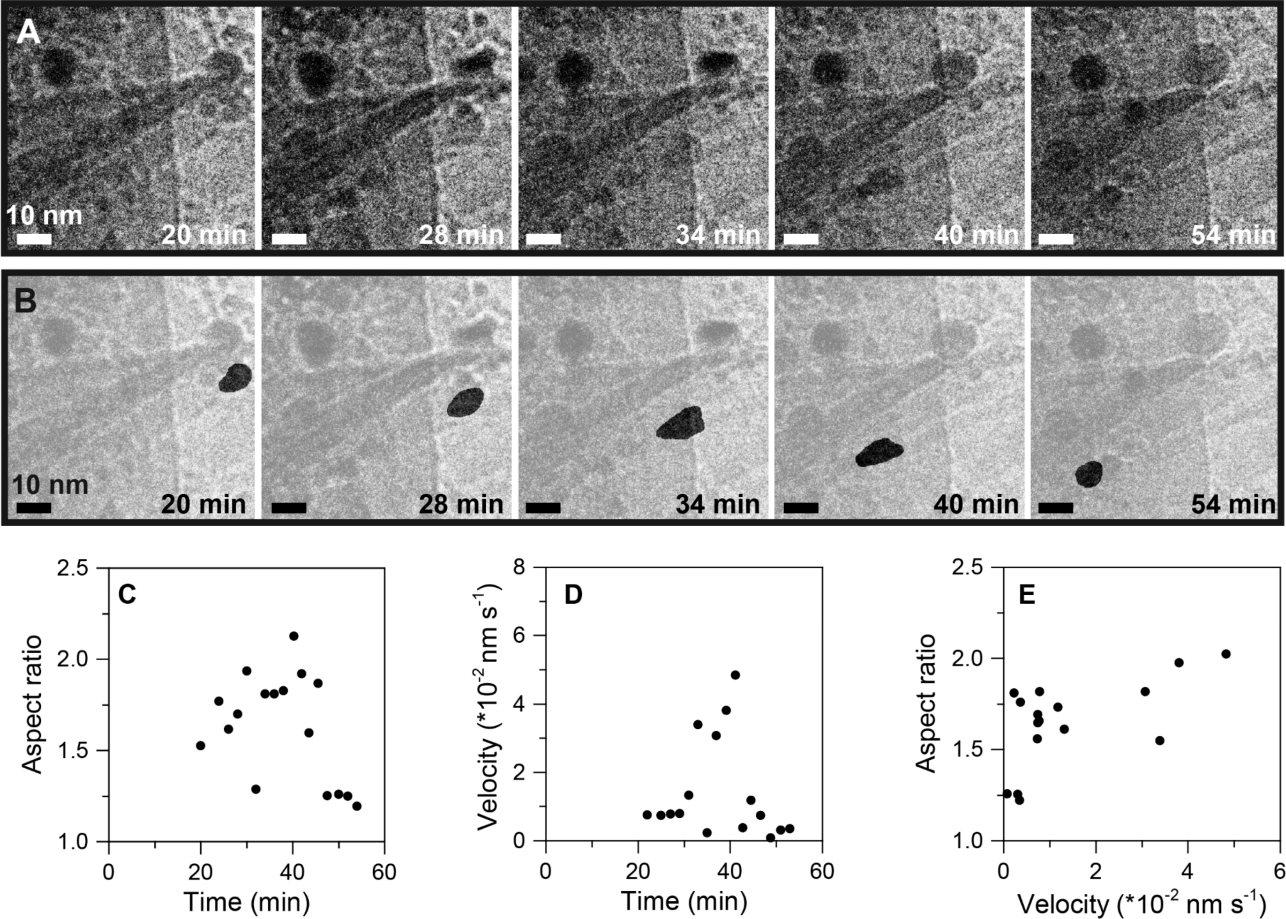
Particle reshaping and movement. (A, top row) TEM images at (from left to right) *t* = 20, 28, 34, 40, and 54 min. (B, middle row) Analysis of the size, position, and shape of an individual particle overlaid on the original EM images. (C) Aspect ratio (*d*_max_/*d*_min_) versus time of an ellipse that was fitted on the mask using ImageJ versus time. (D) Velocity versus time. (E) Aspect ratio versus velocity. During the measurement, the sample was exposed to an electron dose of 8 e^–^ A^–2^ s^–1^ every 2 min for 10 s.

We studied in more detail an example of the slow movement, as illustrated in Figure 4 and Movie S1. Figure 4A shows a TEM time-lapsed series, and in Figure 4B the shape of the particle is overlaid on the original EM images. The full range of images is shown in Figure S12. The velocity of the particle was analyzed as a function of time (Figure 4C). The particle moved into the field of view at *t* = 20 min, and the maximum velocity was about 0.05 nm s^–1^. During its movement, the particle followed the carbon morphology without noticeable interruptions, throughout the entire experiment, which might explain the straight directional movement of this nanoparticle. Interestingly, a high aspect ratio for the nanoparticle, well above 1.0, was observed during a large part of the movement (Figure 4D). Correlating aspect ratio to particle velocity (Figure 4E) shows that the largest aspect ratios were obtained for the highest velocities, which means that when moving fast the particle shape was most elongated. These results suggest that particle shape and particle movement are correlated.

The movement of Ni nanoparticles (Figures 3 and 4) can be attributed to two factors: (i) thermal random movement and (ii) movement due to support gasification. Particle elongation might be caused by the interaction between nickel and carbon, as also observed during growth of carbon nanotubes from CH_4_.^39^ In Figure 4, the path followed by the particle appeared lighter, which is most probably caused by carbon gasification (as Ni is an active methanation catalyst and hydrogen is present in the atmosphere). To confirm this, an *in situ* TEM experiment was performed under conditions very favorable for carbon methanation (H_2_:Ar = 4:1, so replacing the CO_2_ by Ar), indeed resulting in multiple observations of directional particle movement and the subsequent formation of straight channels in the field of view (Figure S13). Under a pure Ar atmosphere, no channel formation was observed with *in situ* TEM, as gasification cannot occur without hydrogen (Figure S4). Our results are in line with previous studies, in which nanoparticle movement over a carbon support, driven by carbon methanation, was reported to result in the formation of straight channels.^54−56^ However, also under CO_2_ hydrogenation conditions, hence in the presence of hydrogen, no channel formation was observed for most moving particles, and random Ni particle movement (both fast and slow) was detected as the main movement mode. Thus, the presence of CO_2_ in the feed suppresses supports methanation-driven movement.

The fast, intermittent movement could be explained by particles hopping over less favorable areas of the support to a more strongly binding support surface site, for example a defect or an (oxygen-containing) surface group present on the support. The presence of surface groups is known to strongly affect metal particle mobility, as shown for Cu nanoparticles during methanol synthesis conditions.^57^ An experiment with nickel nanoparticles on oxidized carbon (GNP-O) showed significantly less nanoparticle mobility and growth compared to Ni/GNP (Movie S4 and Figure S16), confirming the stabilization of Ni nanoparticles using functionalized carbon. This was in line with results reported for high-pressure CO_2_ hydrogenation, where the use of GNP-O limits Ni particle growth.^47^ Additionally, carbon support methanation can provide an extra driving force for particle movement, resulting in more directional movement (Figure 4), which is also affected by the support morphology.

### Growth of Individual Nanoparticles

**Figure 5:**
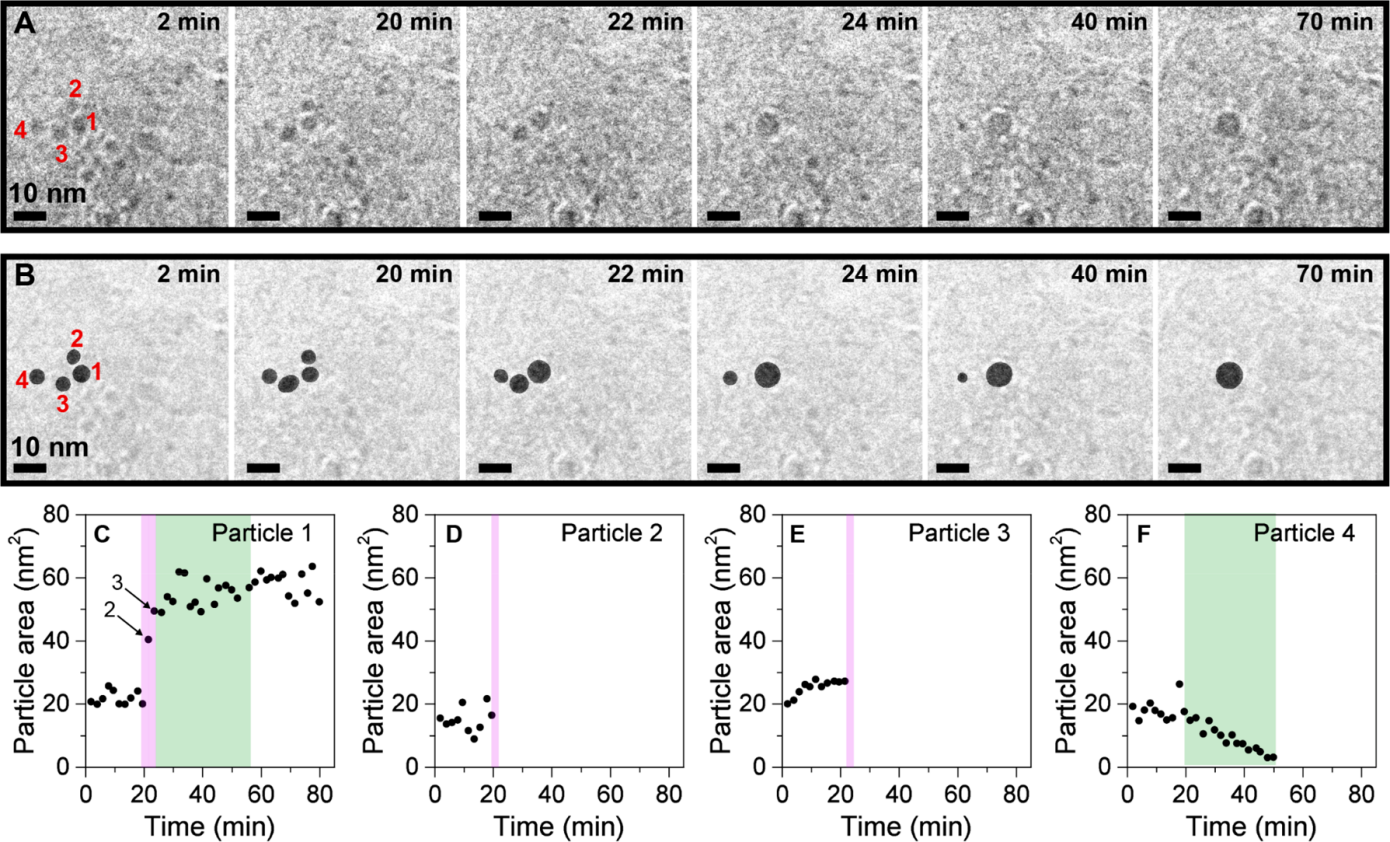
Direct observation of particle growth via coalescence (purple) and Ostwald ripening (green). (A) *In situ* transmission electron microscopy images of Ni/GNP acquired at *t* = 2, 20, 22, 24, and 70 min. (B) Analysis of the projected areas of individual particles overlaid on the original EM images. The bottom row shows the evolution of the projected area of (C) particle 1, where the arrows indicate coalescence with particles 2 and 3, (D) particle 2, (E) particle 3, and (F) particle 4 over time. During the measurement, the sample was exposed to an electron dose rate of 20 e^–^ A^–2^ s^–1^ every 2 min for 10 s.

It is clear from the observations in Figure 1 that the average particle size increases, leading to a decrease in active metal surface area. The particle growth can occur via two mechanisms: (i) particle diffusion and coalescence and (ii) Ostwald ripening. Figure 5 shows a time-lapsed series of four Ni nanoparticles forming one nanoparticle through an interplay of both mechanisms. The snapshots in Figure 5A show the electron microscopy images of the particles at selected times. Figure 5B shows the projected areas of the four particles, analyzed every 2 min over the course of 70 min in total. Figure 5C–F shows the evolution of the projected areas of the four particles over time. Particle 2 (Figure 5D) and particle 3 (Figure 5E) disappear due to coalescence with particle 1, highlighted in the purple regions. This means that at the same time the surface area of the (fused) particle 1 increased stepwise (Figure 5C). The full analysis of the corresponding spherical diameters and particle volumes is displayed in Figure S15. The contribution of other (small) neighboring nanoparticles to the growth of particle 1 cannot be fully excluded. However, this was unlikely to be significant, as the analysis of the particle volumes (Figure S14) shows that the sum of the 4 individual particle volumes (∼250 nm^3^) was similar to the volume of the final particle (∼300 nm^3^). Small variations in measurements were caused by actual changes in area due to reshaping, were due to the translation of a 3D object to a 2D image, or were due to the uncertainties in determining the particle area due to the limited contrast between nickel particles and carbon support. Most coalescence events occurred rapidly, and within one frame, even during continuous imaging every 2 s (Movie S3).

Interestingly, particle 4 showed different growth behavior. During the initial 24 min of the measurement, when the coalescence of particles 1, 2, and 3 took place, particle 4 did not move and the projected area of particle 4 was hardly affected (Figure 5F). There was little difference in the initial size of all four particles, having an area of ∼20 nm^2^_._ However, the distance between particle 1 and 4 was 5.4 nm (*t* = 24 min), which was significantly larger than the distances of 1.2 nm between particles 1 and 2 (*t* = 20 min) and particles 1 and 3 (*t* = 22 min) directly before coalescence took place. The larger interparticle distance between particle 1 and 4 may explain why coalescence did not occur.^12^ However, after the first 20 min in which particle 1 had grown to ∼60 nm^2^, particle 4 started shrinking until it completely disappeared after 50 min (highlighted in the green region in Figure 5F). This sudden change in particle growth behavior from coalescence to Ostwald ripening can be explained by the increased driving force for Ostwald ripening due to the more pronounced size difference between particles 1 and 4.^14,49,58^ Similar events were observed during the various experiments and a second example is shown in Figure S16. Both coalescence and Ostwald ripening resulted in a decrease in the specific Ni surface area. During the entire experiment, the total Ni surface area of the four nanoparticles decreased from 130 to 80 m^2^ g_Ni_^–1^. In this specific example, coalescence contributed more to the decrease in specific surface area than Ostwald ripening (Figure S15P). However, a full statistical study is beyond the scope of this paper. We note that the particle growth likely continues at time scales longer than the measurement time used here (80 min), given the extended time scales of deactivation of these catalysts reported in reactor studies^47^ and the continuous movement of particles during the measurement (e.g., Figure 3F–J).

The interparticle distance seemed less crucial for Ostwald ripening than for coalescence events, in line with observations during catalysis reported in the literature.^49^ Transport of metal species between nanoparticles can take place via surface diffusion or through the gas phase.^8^ Metal evaporation is unlikely to play a role in our experiments, because of the large energy barrier to overcome and the long time it would take to fully evaporate (∼130 h at 750 °C for a 2.5 nm particle).^59^ Diffusion of metal cations stabilized by surface groups and ligands might occur under (slightly) oxidizing circumstances.^60−63^ Nickel could form gaseous Ni(CO)_4_,^49^ with CO formed during the reaction (Table S1). Hence, the formation and decomposition of nickel carbonyl species, controlling Ostwald ripening during CO hydrogenation,^49,58^ might have played a role in our experiments.

The observation of the dual mechanism of diffusion and coalescence and Ostwald ripening highlights the strengths of *in situ* characterization to understand particle growth mechanisms. Even more important, this can have implications for the catalyst stability. As the driving force for Ostwald ripening is the difference in particle sizes, typically Ostwald ripening does not occur for monodisperse samples. However, in this combined mechanism, Ostwald ripening might not be inherently prevented by using a catalyst with a monodisperse nanoparticle size distribution, as suggested previously for a Pt/Al_2_O_3_ catalyst in 5% H_2_/N_2_ at 600–800 °C.^64^ It would be very interesting to study this further for Ni/GNP using a catalyst with monodisperse nanoparticles. As coalescence events are enhanced by collisions of moving particles, minimizing particle movement during catalysis, by ensuring a strong metal–support interaction (influenced by support nature and morphology), is important. This could be accomplished through, for example, support functionalization^47^ or adding stability promoters.^65^ Here, we achieved a dedicated nanoparticle growth study using a realistic nickel-based catalyst under typical CO_2_ hydrogenation conditions. Our *in situ* observations give valuable insights into the interplay between particle diffusion and coalescence, and Ostwald ripening growth mechanisms.

## Conclusions

Various movement and growth mechanisms of a supported Ni catalyst were directly observed under CO_2_ hydrogenation conditions, using *in situ* gas cell TEM at 1 bar and 450 °C. Careful comparison of the averaged particle growth within a nickel on carbon catalyst *in situ* and *ex situ* validated the relevance of the *in situ* obtained results. Two types of particle movement were observed: fast, intermittent movement, and slow, continuous movement, with an order of magnitude difference in overall particle velocities. More continuous directional movement was correlated to an elongated particle shape and support gasification as a driving force, although gasification was suppressed by the presence of CO_2_. Furthermore, both diffusion/coalescence and Ostwald ripening were observed and related to parameters such as interparticle distance and particle size differences. We identified a dual mechanism: large particles were first formed via coalescence, after which fast Ostwald ripening took place with smaller particles nearby. This study emphasizes the wide variety of particle growth and particle movement events occurring under reaction conditions, the complexity of understanding growth models from *ex situ* characterization alone, and the importance of direct, *in situ*, nanoscale observations of ensembles of nanoparticles.

## Methods

### Synthesis of Carbon-Supported Nickel Catalysts

Nickel nitrate hexahydrate (Ni(NO_3_)_2_·6H_2_O, Sigma-Aldrich, ≥97.0%), graphene nanoplatelets (GNP-500, ∼500 m^2^/g surface area, 0.9 mL g^–1^ pore volume, XG Sciences), and nitric acid (HNO_3_, Merck, 65%) were used as received.

The synthesis method used for this study was incipient wetness impregnation. The full synthesis procedure is described in detail in ref (47). In short, 1.5 g of carbon support was dried for 120 min at 170 °C under dynamic vacuum. This carbon was impregnated to incipient wetness with an aqueous Ni(NO_3_)_2_ solution (2 M) under static vacuum while stirring. The sample was dried overnight at room temperature under dynamic vacuum, transferred to a plug-flow reactor, and heated to 350 °C in 200 mL min^–1^ N_2_ (3 °C min^–1^, 90 min) to decompose the nitrate. After cooling down, the sample was reduced in 5% H_2_/N_2_ at 350 °C (200 mL min^–1^, 90 min). Furthermore, GNP was oxidized while stirring in 65% HNO_3_ for 2 h at 80 °C, as described in ref (47), to form GNP-O. The nickel nanoparticles were deposited on GNP-O following the same method as for GNP.

### *Ex Situ* Characterization

*Ex situ* transmission electron microscopy images were taken on a FEI Talos L120C instrument operated at 120 kV. The catalyst sample was dispersed as a dry catalyst powder onto a Cu sample grid coated with holey carbon (Agar 300 mesh Cu).

For complete support and catalyst characterization including X-ray diffraction (XRD), N_2_ physisorption, HR-TEM, and H_2_ chemisorption we refer the reader to ref (47).

### *In Situ* Electron Microscopy Characterization

*In situ* gas-phase TEM measurements were performed on a FEI Talos F200X microscope operated at 200 kV using a Protochips Atmosphere system. Imaging was performed at a magnification of 94 or 120 kx. An objective aperture of 40 μm was used. The cell within the sample holder consisted of a top and bottom chip. An O-ring was used to separate the airtight inner cell compartment from the vacuum of the microscope. The top chip contained a silicon carbide based heating membrane used for closed-loop temperature control using the resistance of the silicon carbide. Both chips contained 30–50 nm thick silicon nitride windows, allowing imaging with the electron beam while containing the gas within the cell. A schematic illustration of the gas cell system is shown in Figure S17. The gas supply system has tanks in which gases can be mixed before they are sent toward the sample holder. All gases were provided by Linde.

The measurements were prepared by drop-casting a sonicated dilute sample dispersed in ethanol onto a plasma-cleaned chip. After mounting, the holder was tested for potential leaks and subsequently inserted in the microscope. Prior to experiments, the lines and the holder were leak checked by evacuating them to 0.1 mbar and monitoring the pressure for 10 min. All leaks were below 0.002 sccm. During the experiments, gases were introduced at 1 bar pressure.

In a typical experiment (temperature and pressure profile illustrated in Figure S18), the holder was pumped down three times to 0.5 mbar and filled with 1000 mbar Ar (purity *N* = 7.0) to flush out from the holder tip. Subsequently the sample was reduced in 5% H_2_/Ar, mixed from pure H_2_ and Ar, both with 7.0 purity, at 0.2 sccm flow for at least 30 min at 300 °C and 1 bar prior to any exposure to the beam. This step was important not only to reduce the passivated Ni(O) particles but also to remove water from the holder and the support surface. The removal of remaining traces of water before exposure to the electron beam was crucial to prevent support damage due to the formation of radicals.^33,66,67^ Subsequently the chip was cooled down to 150 °C and briefly exposed to the electron beam to find suitable areas for imaging and perform alignment. The beam was then blanked and the gas was switched to 4:1 H_2_/CO_2_, which was mixed in the manifold using pure CO_2_ (purity *N* = 5.3) and pure H_2_ (purity *N* = 7.0), with a flow rate of 0.4 sccm. The sample was heated to 400 °C (0.1 °C s^–1^, 40 min) and subsequently heated further to 450 °C (0.1 °C s^–1^, 60–80 min). At 450 °C the nanoparticle growth process was accelerated, allowing for the possibility to follow changes during 1 h of measuring time. For the data analysis, the moment 450 °C was reached was set as *t* = 0.

Three *in situ* experiments were performed. During the first experiment, an image was taken every 2 min and the sample was exposed for approximately 10 s to the electron beam with an electron dose of 8 e^–^ A^–2^ s^–1^_._ These 10 s of beam exposure were used to focus and take an image with an integration time of 2 s. Between the measurements, the beam was blanked. Immediately after the experiment, the temperature was lowered to 25 °C and another location on the grid was found which had not previously been exposed to the beam to compare beam-exposed and nonexposed regions. This experiment was repeated with an electron dose rate of 20 e^–^ A^–2^ s^–1^ to increase the resolution. Finally, after establishing the absence of beam effects at both dose rates, the experiment was performed with continuous exposure to the electron beam (20 e^–^ A^–2^ s^–1^). In this case the sample was also continuously imaged, keeping an integration time of 2 s.

### Validation by Comparison with *Ex Situ* Measurement

Validation tests for the *in situ* TEM experiments were performed in the *in situ* TEM holder without beam exposure and in a high-throughput gas-phase 16-parallel fixed bed reactor system (Avantium Flowrence). For the first, a typical gas cell experiment was repeated with the holder in a vacuum pump station instead of in the microscope. The gas and temperature procedures were the same as during the *in situ* experiments. To avoid beam effects by the simultaneous exposure of the sample to the electron beam and a gas, after each step the holder was disassembled at *t* = −8 min, after the 40 min at 400 °C under 1 bar CO_2_/H_2_ (see Figure S18), and the chip containing the sample was analyzed in a TEM inspection holder under vacuum. Subsequently the holder was reassembled and the experiment was continued. At the end of the experiment (*t* = 52 min), the holder was disassembled again and the same areas of the chip were imaged in the inspection holder.

To check the catalytic activity under the reaction conditions used for the *in situ* experiments, 0.5 mg of catalyst sieved in a fraction of 38–75 μm was loaded on top of ∼0.5 cm SiC granules and topped off with SiC. A reduction step was performed in an 11 mL min^–1^ flow of 10% H_2_/N_2_ at 300 °C (10 °C min^–1^) for 30 min. Then the reactor was cooled down to 140 °C and the gas was switched to a mixture of H_2_:CO_2_:He with a ratio of 76:19:5 and a flow of 12 mL min^–1^ per reactor at 1 bar, resulting in a GHSV of 1.3 × 10^6^ mL g_cat_ h^–1^. He is typically used in the catalytic setup as a reference gas. After ensuring a stable gas composition for 30 min, the temperature was increased to 400 °C at a rate of 10 °C min^–1^ and this temperature was held for 40 min. Subsequently the temperature was further increased to 450 °C with a ramp of 6 °C min^–1^. This was held for 52 min (the total time including the final heating step was 60 min). The products were analyzed directly with online gas chromatography (GC, Agilent 7890B) with a sampling time of 14 min. In a test with the same procedure, the CO_2_ content was replaced by N_2_, to investigate roughly how much CH_4_ formed could be attributed to support methanation.

The stainless steel reactor tubes used in this setup have a diameter of 2.6 mm, large enough to accommodate the silicon nitride gas cell bottom window chips (2 × 2 mm chips). Hence, the *in situ* TEM measurement could be repeated in the catalytic setup under similar conditions, which was done with two bottom window chips in two separate reactors. For this purpose, the reactor tubes were first loaded with 1–2 cm long hollow glass wool filaments, in order to ensure that the chips were placed in the isothermal zone. The chips with drop-casted catalyst were placed on top of the glass wool filament in the reactor. The same gas conditions (containing H_2_, CO_2_, and He) and temperature profile were used as described in the previous paragraph. After the test, the reactors were flushed with He and cooled down to remove the chips from the reactor to analyze with TEM. The GHSV during these experiments was 7.1 × 10^4^ mL_gas_ mL_reactor_^–1^ h^–1^, taking into account only the reactor volume that actually contained the TEM chip of 2 mm in height, compared to 2.4 × 10^4^ mL_gas_ mL_tip_ h^–1^ during *in situ* TEM, assuming a holder tip volume of 1 μL.

### Data Analysis

The data were analyzed using ImageJ software. After carefully aligning the images, a field of view was selected. At time intervals of 10 min, the projected area of all nanoparticles in the field of view was determined. We measured nanoparticle area (nm^2^) instead of volume, as TEM is a two-dimensional technique. An elliptical shape was used to represent the projected area of the nanoparticles, adapting the aspect ratio of the ellipse to match the shape of the particles. ImageJ software calculated the projected area of each measured particle, which was used for further analysis.

The averaged area (*A*_n_, nm^2^) was calculated as
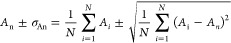
1where *N* indicates the total number of measured particles and *A*_*i*_ stands for the area of the *i*th particle.

From this, particle diameter of each individual particle was calculated as follows, assuming a spherical shape
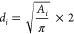
2where *d*_*i*_ indicates diameter of the *i*th particle (nm). From these particle diameters, the number-averaged particle diameter (*d*_n_) of the nickel particles including the standard deviation was calculated as follows:
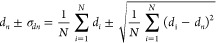
3For each nanoparticle, the volume was *v*_*i*_ (nm^3^) calculated from the spherical particle diameter, and subsequently the amount of nickel per particle
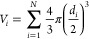
4
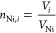
5where *V*_Ni_ is the volume of a nickel atom (0.01095 nm^3^).^68^

The amount of surface Ni was calculated per particle via the dispersion (*D* (%) = 1/d_*n*,*i*_):^68^
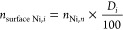
6Finally, the specific metal surface area (m^2^ g_Ni_) was calculated as follows
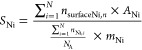
7Where A_Ni_ is the area occupied by a Ni surface atom (0.0951 nm^2^),^68^ N_A_ is 6.022 × 10^23^ mol^–1^ and m_Ni_ is the molar mass of Ni (58.6934 g mol^–1^).

The velocity of the particle movement (nm s^–1^) was analyzed using the equation
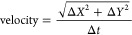
8where *X* and *Y* indicate the center of mass coordinates of the particle. To plot the velocity as a function of time, the average between two time intervals (Δ*t*) was used on the *x* axis.

For the analysis of the particle shape in Figure 4, an ellipse was fitted to the projected area of each particle, and the maximum (*d*_max_) and minimum diameters (*d*_min_) were extracted from this fitted ellipse. The particle shape was expressed as the aspect ratio using the following equation:
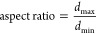
9To plot the aspect ratio as a function of velocity (Figure 4F), the average aspect ratio between two time intervals was calculated and plotted on the *y* axis.

## Supporting Information Available

Catalytic results, details about the validation of the electron beam dose used and the *ex situ* comparisons. effect of different gas atmospheres, additional analysis of particle movement and growth, illustration of the gas cell holder, and experimental procedures (PDF)
Complete area imaged during *in situ* exposure to H_2_:CO_2_ = 4:1, 450 °C, 1 bar and an e^–^ beam dose of 8 e^–^ A^–2^ s^–1^ for 10 s every 2 min, highlighting the area analyzed in Figure 1 and the particle analyzed in Figure 4 (MPG)
Complete area imaged during *in situ* exposure to H_2_:CO_2_ = 4:1, 450 °C, 1 bar and an e^–^ beam dose of 20 e^–^ A^–2^ s^–1^ for 10 s every 2 min, highlighting the particles analyzed in Figure 5 (MPG)
Complete area imaged during *in situ* exposure to H_2_:CO_2_ = 4:1, 450 °C, 1 bar and a continuous e^–^ beam dose of 20 e^–^ A^–2^ s^–1^, highlighting the particles analyzed in Figure 3 (MPG)
Complete area of nickel on *oxidized* carbon during *in situ* exposure to H_2_:CO_2_ = 4:1, 450 °C, 1 bar and an e^–^ beam dose of 8 e^–^ A^–2^ s^–1^ for 10 s every 2 min, highlighting the area analyzed in Figure S14 (MPG)

## Supplementary Material

nn3c03721_si_001.pdf

nn3c03721_si_002.mpg

nn3c03721_si_003.mpg

nn3c03721_si_004.mpg

nn3c03721_si_005.mpg
