# Supplementary material for: Direct Observation of Ni Nanoparticle Growth in Carbon-Supported Nickel under Carbon Dioxide Hydrogenation Atmosphere
Source: ACS Nano. 2023 Jul 28;17(15):14963–73. doi: 10.1021/acsnano.3c03721 (PMC10416566; doi:10.1021/acsnano.3c03721)
Supplement: Supplementary file 1 — nn3c03721_si_001.pdf [file nn3c03721_si_001.pdf]

Supporting information

Direct observation of Ni nanoparticle growth in carbon supported nickel under carbon dioxide hydrogenation atmosphere

Nienke L. Visser,[a] Savannah J. Turner,[a] Joseph A. Stewart,[c] Bart D. Vandegehuchte,[c] Jessi E.S. van der Hoeven,[a*] Petra E. de Jongh[a*]

[a] Materials Chemistry and Catalysis, Debye Institute for Nanomaterials Science, Utrecht University, Universiteitsweg 99, 3584 CG, Utrecht, The Netherlands

[c] TotalEnergies OneTech Belgium, B-7181 Seneffe, Belgium

* Corresponding authors: P.E.deJongh@uu.nl and J.E.S.vanderHoeven@uu.nl

Table of Contents

Section S1. Catalytic results..2

Section S2. Exposure to 8 e^{-} A^{-2} s^{-1} for 10 s every 2 min..3

Section S3. Exposure to pure Ar..4

Section S4. Overview beam damage check..5

Section S5. Exposure to 20 e^{-} A^{-2} s^{-1} for 10 s every 2 min..6

Section S6. Continuous exposure to 20 e^{-} A^{-2} s^{-1}..7

Section S7. *Ex situ* comparisons..9

Section S8. Additional analysis particle movement..10

Section S9. Additional analysis particle growth..13

Section 10. Additional figures for experimetal methods..14

Section 11. Supporting references..15

Section S1. Catalytic results

Table S1. Results from catalytic test showing the GC peak concentrations of inert SiC and of 0.5 mg catalyst (sieve fraction of 38-75 µm) during exposure to CO_2 hydrogenation conditions at 450 °C and 1 bar (top rows) and in the absence of CO_2 (replaced by N_2, bottom rows). Even at the high GHSVs used, CO_2 was converted to CO (main product) and CH_4. In absence of CO_2, the CH_4 concentration was only <1% compared to the test where CO_2 was present, showing that during CO_2 hydrogenation, the formed CH_4 mainly originated from CO_2 conversion instead of support methanation.

Gas composition	Sample	GHSV	Catalyst loading	[He]	[CO_2]	[CH_4]	[CO]	CO_2 conversion
		(* 10^6 mL g_{cat}^{-1} h^{-1})	(mg)	(%)	(%)	(%)	(%)	(%)
H_2:**CO_2**:He 76:19:5	SiC	-	-	4.404	17.137	0	0.022	-
	Ni/GNP	1.3	0.58	4.453	13.341	0.301	3.518	23
H_2:**N_2**:He 76:19:5	SiC	-	-	4.379	0	0	0.001	-
	Ni/GNP	1.4	0.53	4.383	0	0.002	0	-

Section S2. Exposure to 8 $e^- A^{-2} s^{-1}$ for 10 s every 2 min

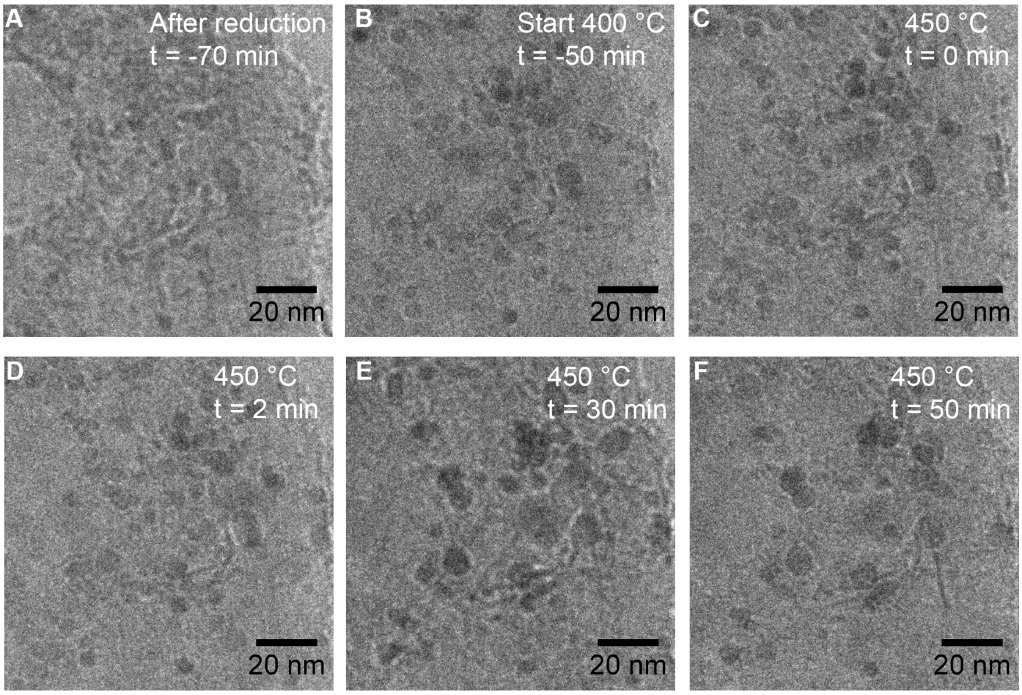

Figure S1. TEM images of the data shown in Figure 1 before the start of the actual experiment: A) Directly after in situ reduction of the sample at 300 °C (5% H_2/Ar). Subsequently the gas was switched to H_2:CO_2 = 4. **B)** After heating the sample to 400 °C, **C)** After heating the sample to 450 °C. **D-F**) TEM images as also shown in Figure 1A-C without highlights of the nanoparticles. For the details about the exact protocol indicating the different times, we refer to Figure S18.

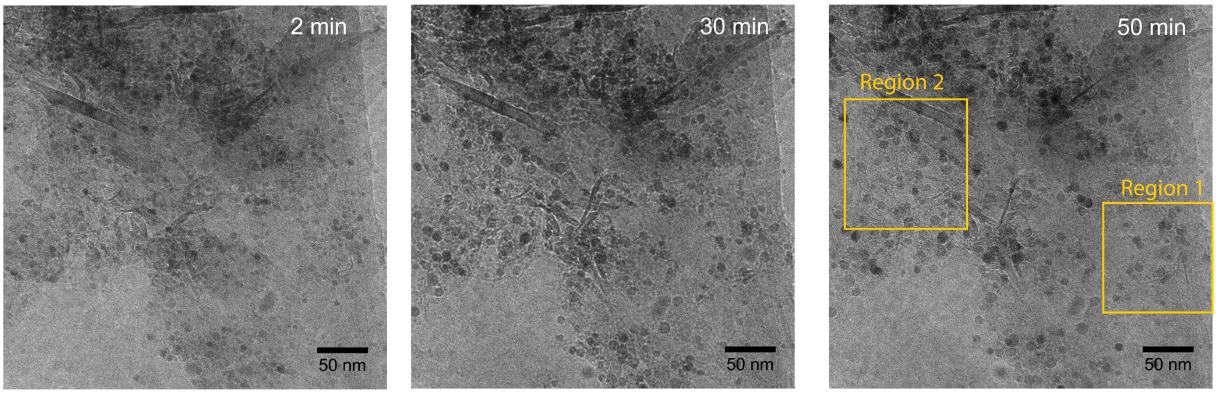

Figure S2. TEM images at different moments in time of the full region of Ni/GNP during the experiment where the imaged areas were exposed 1 bar, 450 °C, 0.4 sccm, 4:1 H_2:CO_2 and an electron dose of 8 $e^- s^{-1} A^{-2}$ every 2 min for ~10 s. The two analyzed regions are highlighted (the analysis of region 1 is depicted in Figure 1 and of region 2 in Figure S2).

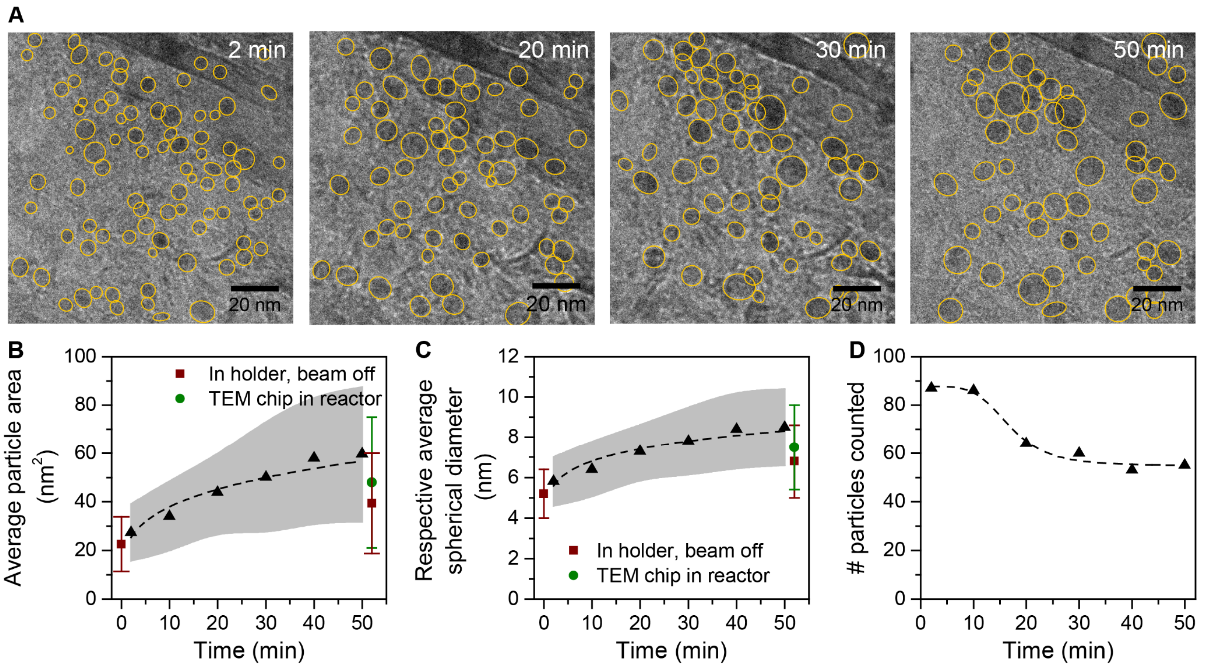

Figure S3. Analysis of region 2 in Figure S2. **A)** Transmission electron microscopy images of Ni/GNP acquired after *t* = 2, 20, 30 and 50 min. The perimeter of all observed particles is highlighted in yellow. **B)** Average projected particle area and **D)** average respective particle diameter calculated from the projected particle areas assuming spherical shape as a function of time. In grey the standard deviation of the measurements is shown. To investigate beam effects, the experiments were repeated on a TEM chip the *in situ* TEM holder without exposure to the beam (red squares) and on a TEM chip in a fixed bed reactor (green circles). **D)** Total number of particles in field of view. All dashed lines are added as guide for the eye. During the experiment, the imaged areas were exposed 1 bar, 450 °C, 0.4 sccm 4:1 H_2:CO_2 and an electron dose of 8 $e^- s^{-1} A^{-2}$ every 2 min for ~10 s.

Section S3. Exposure to pure Ar

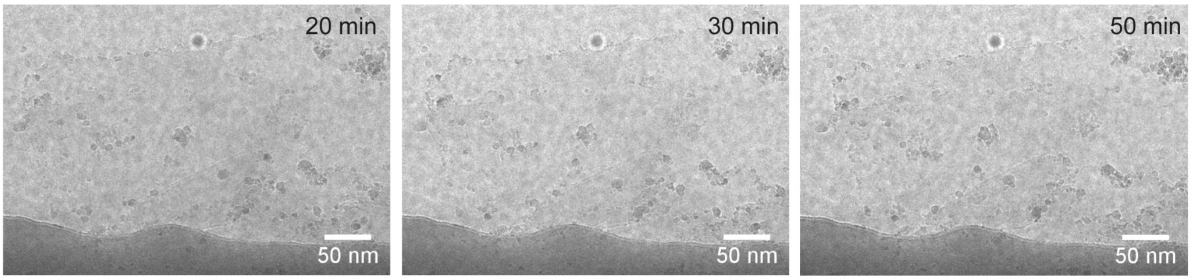

Figure S4. TEM images under pure argon flow at different moments in time of Ni/GNP during the experiment where the imaged areas were exposed 1 bar, 450 °C, 0.4 sccm, Ar and an electron dose of 20 $e^- s^{-1} A^{-2}$ every 2 min for ~10 s. Barely any particle growth was observed during this experiment, however the support was not stable. This was possibly caused by traces of oxygen-containing contaminations, burning the carbon and feasibly oxidizing the nanoparticles, the formation of a mild plasma under the electron beam[1], or remaining presence of water causing edging of the outer layers of the carbon structures under electron irradiation.[2]

Section S4. Overview beam damage check

Table S2. Overview of all *in situ* and *ex situ* TEM experiments, showing average particle diameter (d) and number of particles in field of view at start and end performed. All experiments were performed at 450 °C, 1 bar H_2:CO_2 = 4:1. For the exact details of each experiment, a referral to the corresponding figures with the data analysis is given in the table.

Figure	Beam exposure	Beam dose	Field of view	0 - 2 min		50-52 min	
				$d \pm \sigma$	# particles	$d \pm \sigma$	# particles
		(e^{-} s^{-1} A^{-2})	(nm x nm)	(nm)		(nm)	
1	~10 s Every 2 min	8	108 x 109	5.7 ± 1.4	72	7.7 ± 1.9	34
S3	~10 s Every 2 min	8	119 x 122	5.8 ± 1.2	87	8.5 ± 1.9	55
S5	~10 s Every 2 min	20	107 x 107	4.2 ± 1.1	61	6.2 ± 1.7	45
S7 (top)	Continuous	20	119 x 108	5.7 ± 1.3	47	6.3 ± 1.5	30
S7 (bottom)	Continuous	20	100 x 100	5.1 ± 1.0	72	6.9 ± 1.7	50
S8	None (holder)	-		5.2 ± 1.2	365	6.8 ± 1.8	199
S9	None (reactor)	-		-	-	7.5 ± 2.1	370

Table S3. Overview of all *in situ* and *ex situ* TEM experiments, showing average (*d*), de median and the geometric mean particle diameters at the start and the end of the experiment. All experiments were performed at 450 °C, 1 bar H_2:CO_2 = 4:1. For the exact details of each experiment, the corresponding figures are indicated in the table.

Figure	0 - 2 min			50 - 52 min		
	$d \pm \sigma$	Median	Geometric mean	$d \pm \sigma$	Median	Geometric mean
		(nm)			(nm)	
1	5.7 ± 1.4	5.5	5.6	7.7 ± 1.9	7.7	7.4
S3	5.8 ± 1.2	5.7	5.7	8.5 ± 1.9	8.0	8.3
S5	4.2 ± 1.1	4.4	4.4	6.2 ± 1.7	6.2	6.0
S7 (top)	5.7 ± 1.3	5.6	5.6	6.3 ± 1.5	6.1	6.1
S7 (bottom)	5.1 ± 1.0	5.2	5.1	6.9 ± 1.7	6.4	6.7
S8	5.2 ± 1.2	5.3	5.3	6.8 ± 1.8	6.7	6.6
S9	-	-	-	7.5 ± 2.1	7.3	7.2

Section S5. Exposure to 20 e^- A^{-2} s^{-1} for 10 s every 2 min

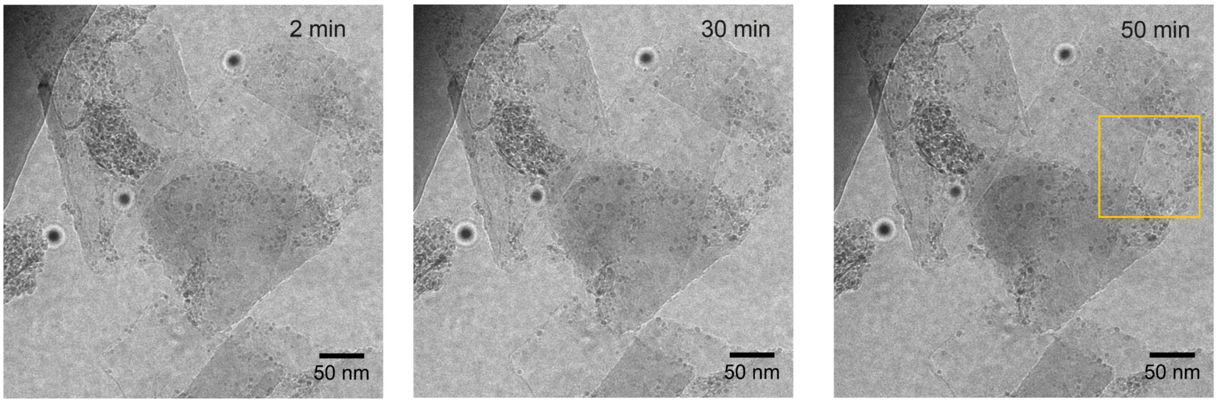

Figure S5. TEM images at different moments in time of the full region of Ni/GNP during the experiment where the imaged areas were exposed 1 bar, 450 °C, 0.4 sccm, 4:1 H_2:CO_2 and an electron dose of 20 e^- s^{-1} A^{-2} every 2 min for ~10 s. The two analyzed region is highlighted and the analysis is depicted in Figure S6.

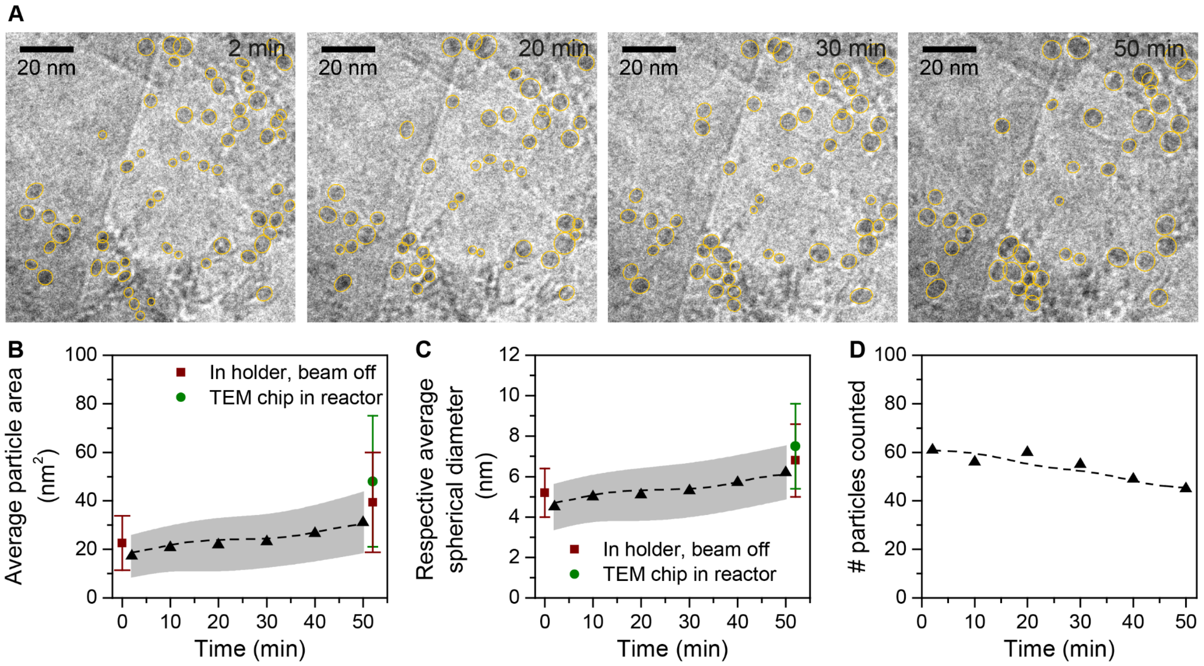

Figure S6. Analysis of the highlighted region in Figure S5. **A)** Transmission electron microscopy images of Ni/GNP acquired after *t* = 2, 20, 30 and 50 min. The perimeter of all observed particles is highlighted in yellow. **B)** Average projected particle area and **D)** average respective particle diameter calculated from the projected particle areas assuming spherical shape as a function of time. In grey the standard deviation of the measurements is shown. To investigate beam effects, the experiments were repeated on a TEM chip the *in situ* TEM holder without exposure to the beam (red squares) and on a TEM chip in a fixed bed reactor (green circles). **D)** Total number of particles in field of view. All dashed lines are added as guide for the eye. During the experiment, the imaged areas were exposed 1 bar, 450 °C, 0.4 sccm 4:1 H_2:CO_2 and an electron dose of 20 $e^- s^{-1} A^{-2}$ every 2 min for ~10 s.

Section S6. Continuous exposure to 20 $e^- A^{-2} s^{-1}$

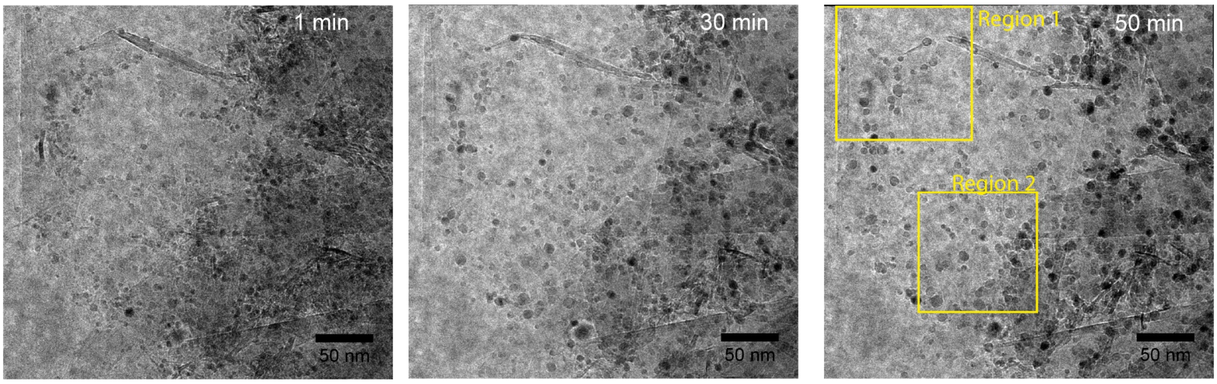

Figure S7. TEM images at different moments in time of the full region of Ni/GNP during the experiment where the imaged areas were exposed 1 bar, 450 °C, 0.4 sccm, 4:1 H_2:CO_2 and a continuous electron dose of 20 $e^- s^{-1} A^{-2}$. The analysis of the two highlighted regions is shown in Figure S8.

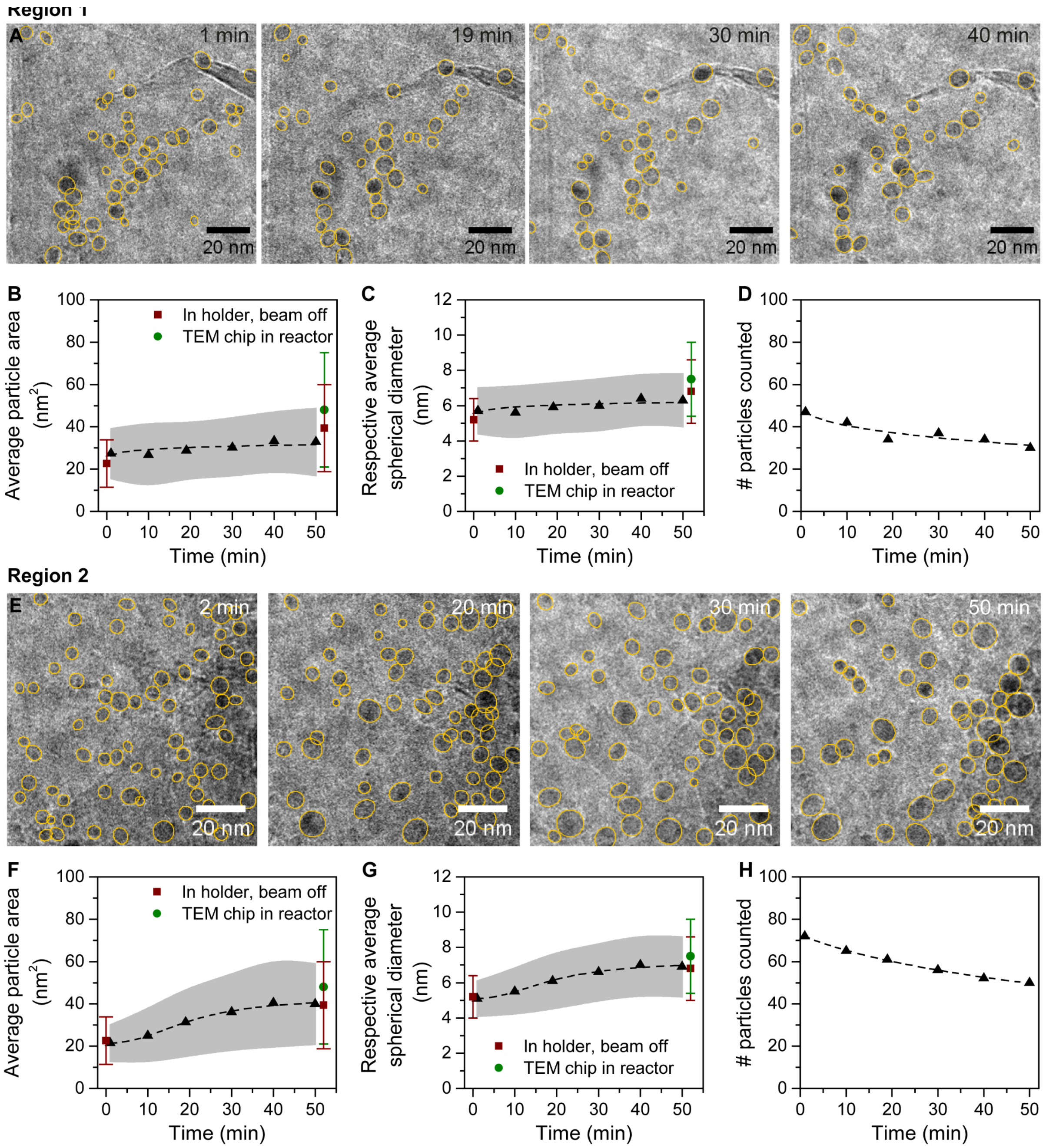

Figure S8. Analysis of the highlighted regions in Figure S7. **A,E)** Transmission electron microscopy images of Ni/GNP acquired after *t* = 2, 20, 30 and 50 min. The perimeter of all observed particles is highlighted in yellow. **B,F)** Average projected particle area and **D,G)** average respective particle diameter calculated from the projected particle areas assuming spherical shape as a function of time. In grey the standard deviation of the measurements is shown. To investigate beam effects, the experiments were repeated on a TEM chip the *in situ* TEM holder without exposure to the beam (red squares) and on a TEM chip in a fixed bed reactor (green circles). **D,H)** Total number of particles in field of view. All dashed lines are added as guide for the eye. During the experiment, the imaged areas were exposed 1 bar, 450 °C, 0.4 sccm 4:1 H_2:CO_2 and a continuous electron dose of 20 $e^- s^{-1} A^{-2}$.

Section S7. *Ex situ* comparisons

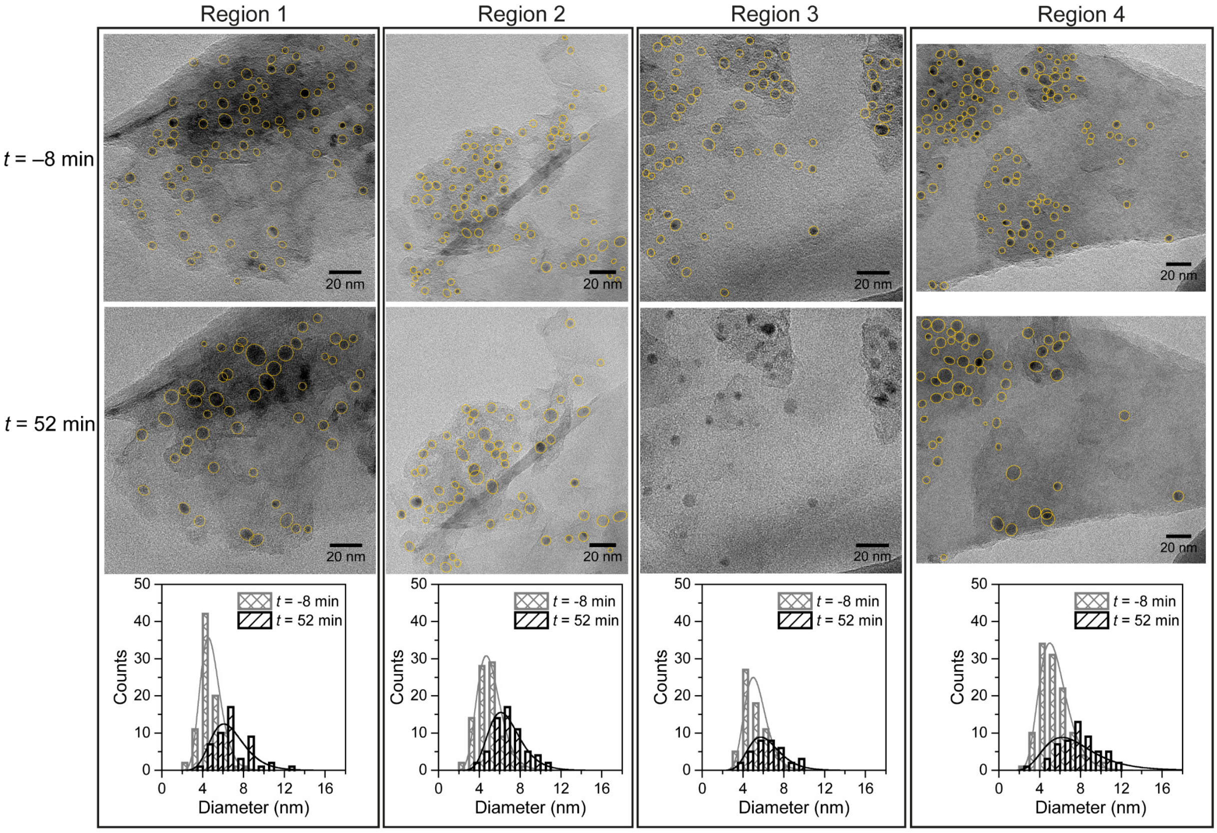

Figure S9. Beam damage check in the *in situ* TEM holder without exposure to the beam. The gas and temperature procedures were the same as during the *in situ* experiments. To avoid beam effects any exposure of the sample to the electron beam and a gas at the same time, after each step the holder was disassembled at *t* = -8 min, after the 40 min at 400 °C under 1 bar CO_2/H_2 (see Figure S18) and the chip containing the sample was analyzed in a TEM inspection holder under vacuum (top row). Subsequently the holder was reassembled and the experiment was continued. At the end of the experiment (*t* = 52 min), the holder was disassembled again and the same areas of the chip were imaged in the inspection holder (middle row). The bottom row shows the histograms of the counted nanoparticles. The results of the 4 regions were used to determine the average particle diameter that is shown in Figure 2 in the manuscript and Table S2.

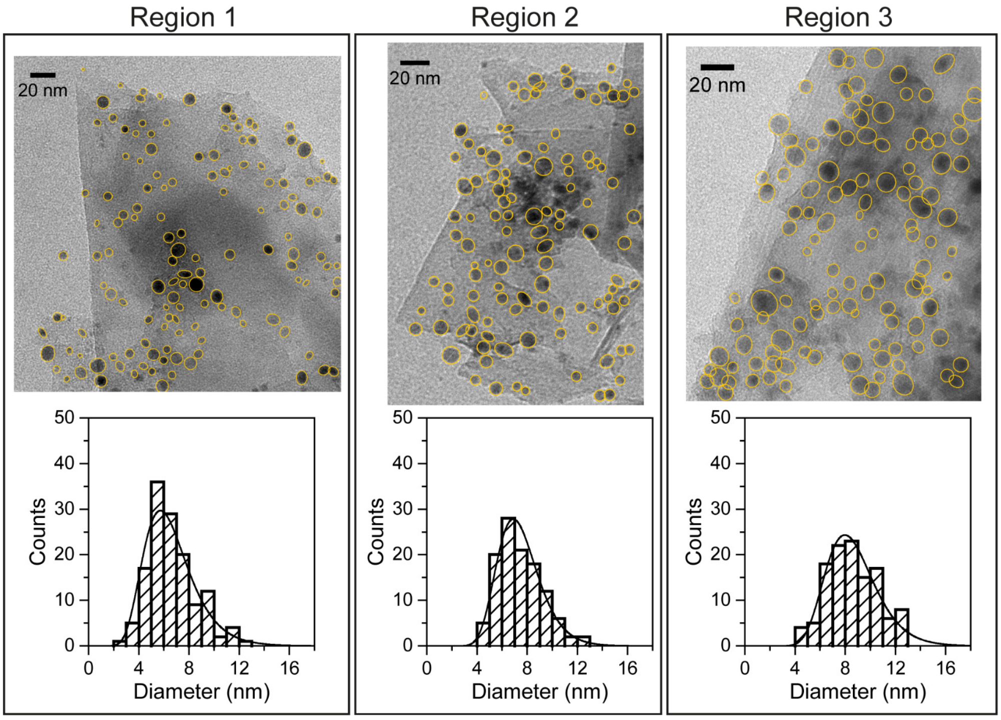

Figure S10. Beam damage check with a TEM chip in a fixed bed reactor setup exposed to a flow of H_2:CO_2:He = 76:19:5 with a GHSV of 7.1 * 10^4 mL_{gas} $mL_{reactor}^{-1}$ h^{-1} at 1 bar and 450 °C. The TEM images show various analyzed regions at the end of the experiment with their corresponding size distribution. The results of these 3 regions were used to determine the average particle diameter that is shown in Figure 2 in the manuscript and Table S2.

Section S8. Additional analysis particle movement

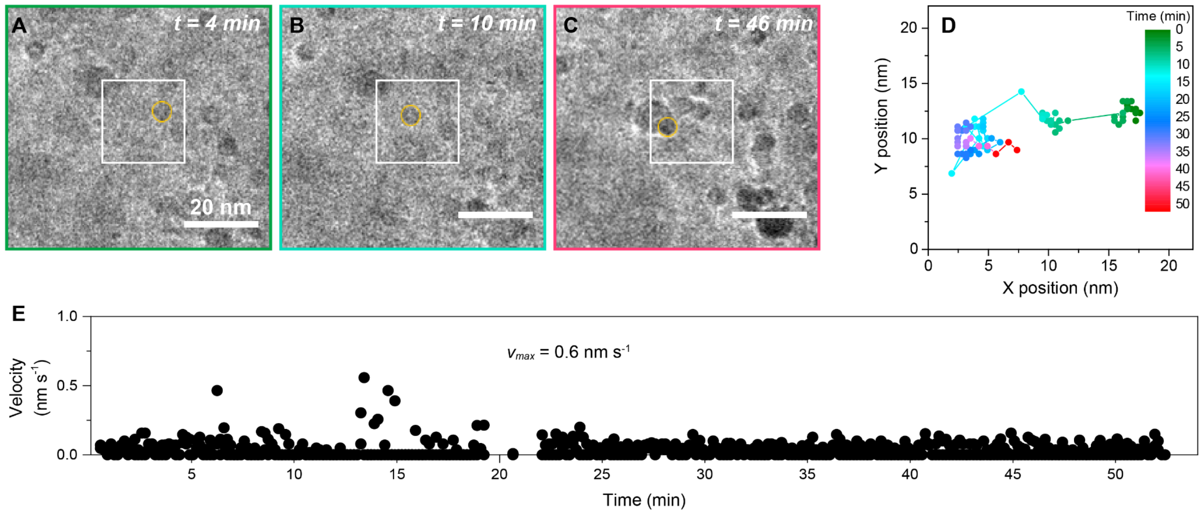

Figure S11. Extra example of particle movement studied *in situ*. TEM images highlight the analyzed particle (top panel) at **A)** t = 4 min, **B)** t = 10 min and **C)** t = 46 min. The white box indicates the frame used to determine the X and Y location of the nanoparticles. **D)** Tracks of the center of the analyzed nanoparticles in the corresponding row. **E)** Velocity versus time of the nanoparticles in the corresponding panel. During the experiment, the imaged area was continuously exposed to an electron dose of 20 e^- A^{-2} s^{-1}. This particle moved via both the fast hopping mechanism and later the slower movement.

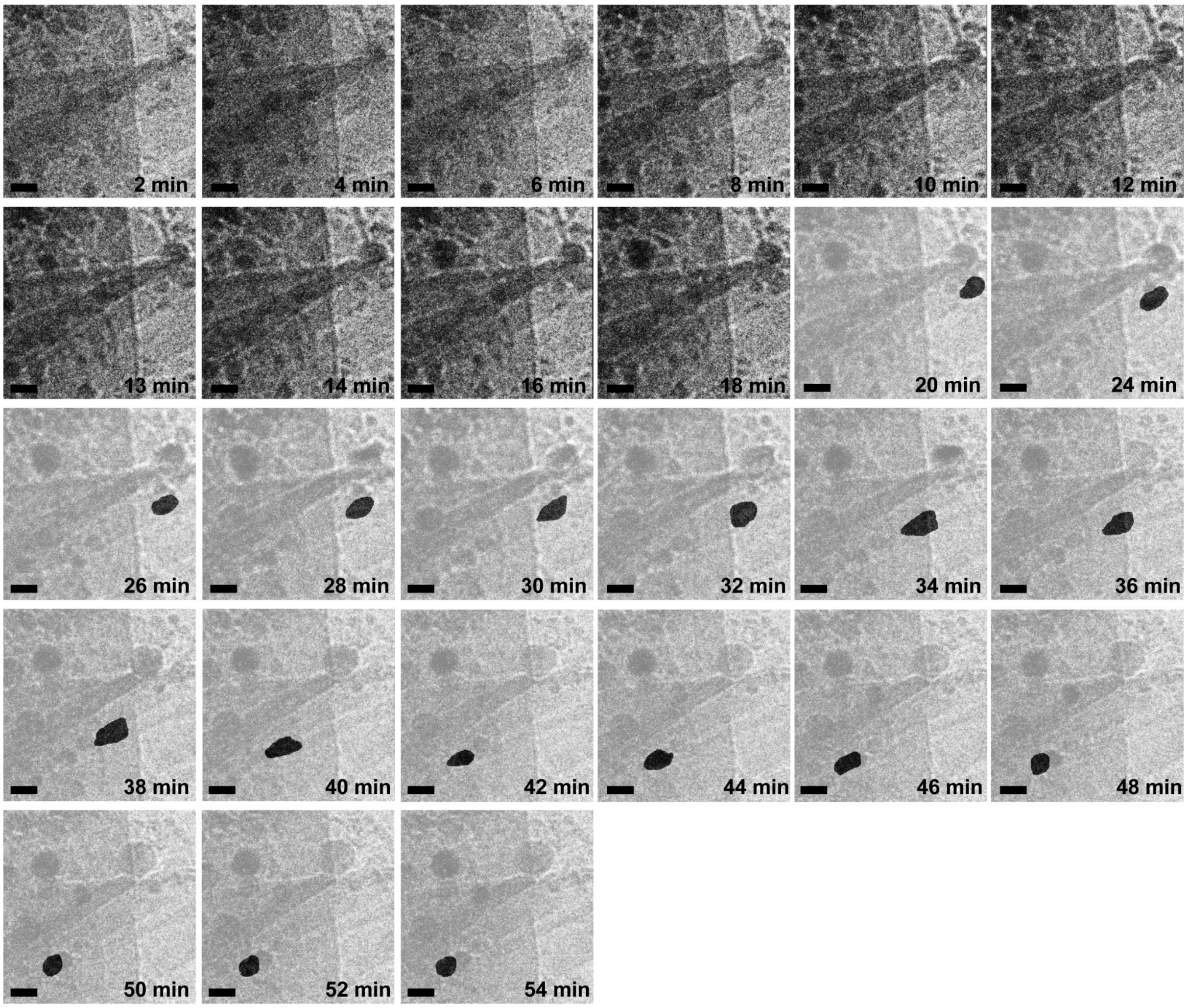

Figure S12. Full time lapse of the data shown in Figure 4. The overlaying mask is shown from the moment the analyzed particle moved into the field of view.

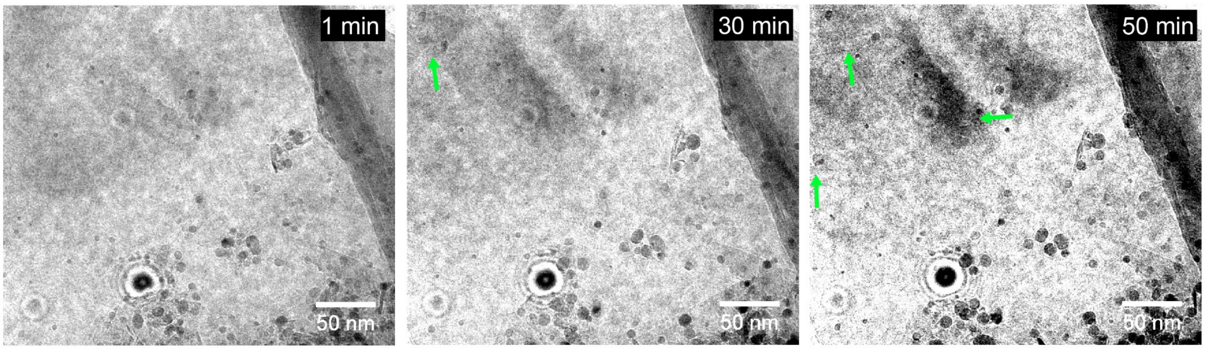

Figure S13. Transmission electron microscopy images of Ni/GNP during *in situ* experiment at 450 °C under 1 bar, 0.4 sccm H_2/N_2 = 4:1 flow, with continuous exposure to an electron dose of 20 $e^- A^{-2} s^{-1}$. The green arrows point to the channels formed in the carbon support due to support methanation.

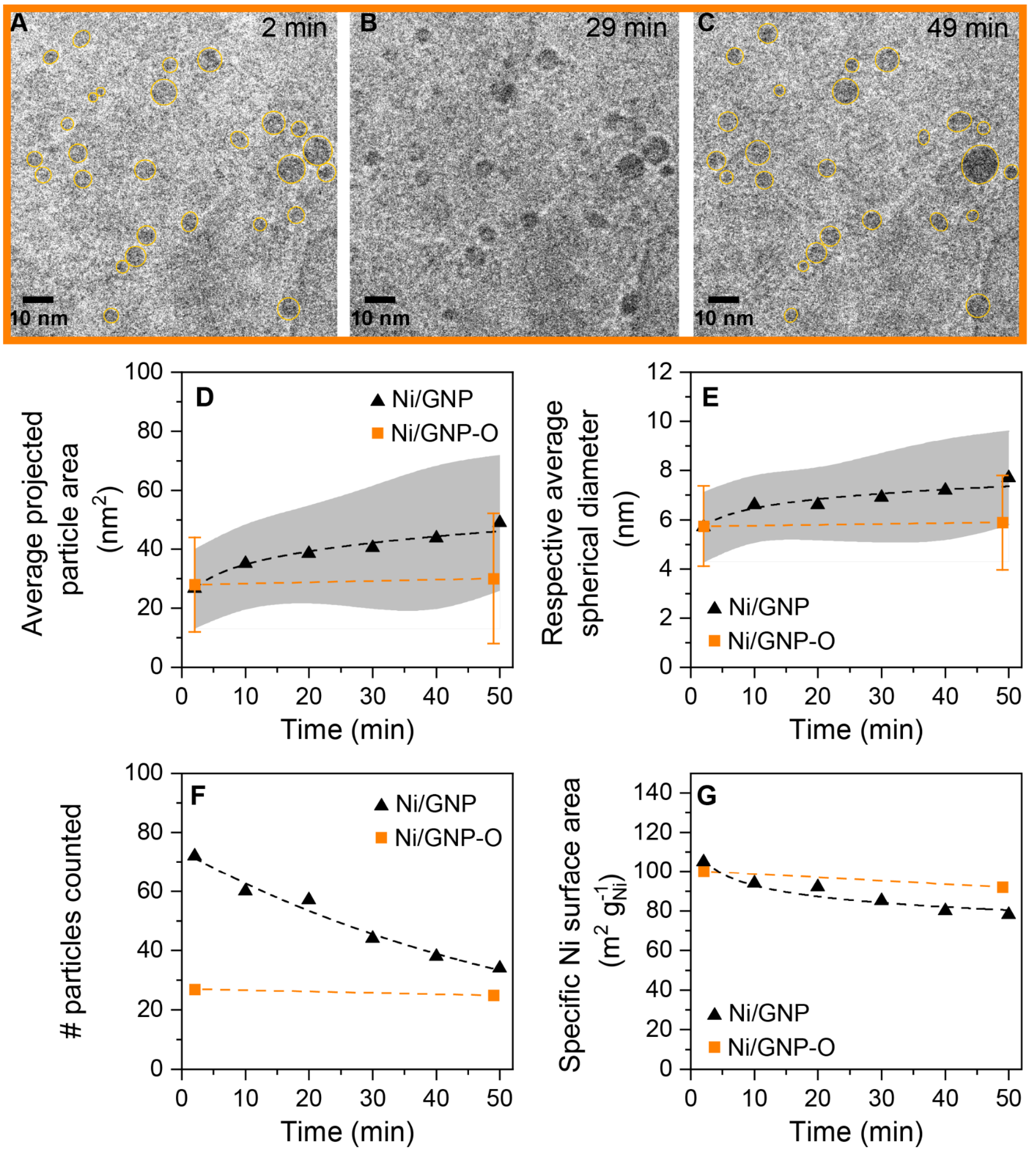

Figure S14. Comparison of particle growth of nickel nanoparticles on pristine carbon (Ni/GNP) and on functionalized (oxidized) carbon (Ni/GNP-O). The data in black is the data shown in Figure 1 (Ni/GNP), whereas the data in orange is the data from the experiment with Ni/GNP-O. Transmission electron microscopy images of Ni/GNP-O acquired after **A)** 2, **B)** 29 and **C)** 49 min. The perimeter of all observed particles is highlighted in yellow. **D)** Average projected particle area and **E)** average particle diameter calculated from the projected particle areas assuming a spherical shape, as a function of time. In grey the standard deviation of the measurements is shown. **F)** Total number of particles in field of view and **G)** specific metal surface area as function of time. Dashed lines are added as guide to the eye. During the experiment, the imaged areas were exposed to an electron dose of 8 $e^- s^{-1} A^{-2}$ every 2 min for ~10 s 450 °C, H_2:CO_2 = 4 and a flow of 0.4 sccm.

Section S9. Additional analysis particle growth

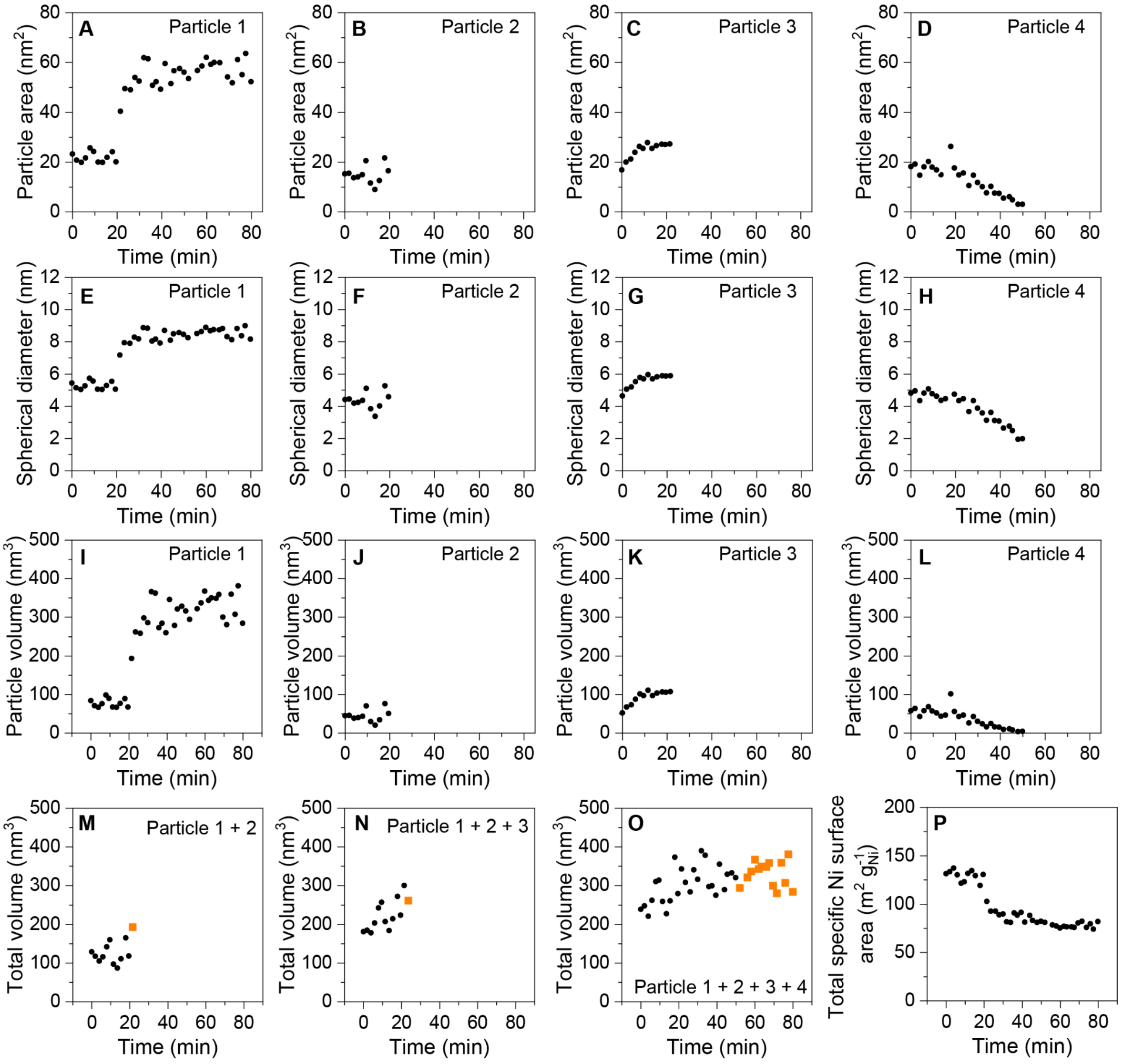

Figure S15. Full analysis of the four nanoparticles shown in Figure 5. **A-D**) Areas, **E-H**) corresponding particle diameters assuming spherical nanoparticles and **I-L)** Particle volumes calculated from the diameters of the individual nanoparticles as function of time. **M-O**) Sum of the volumes of **M**) particles 1 + 2, **N**) particles 1 + 2 + 3 and **O**) all particles as function of time. The orange squares highlight the volume of particle 1 *after* merging of the particles. **P**) Total specific Ni surface area of the four particles versus time.

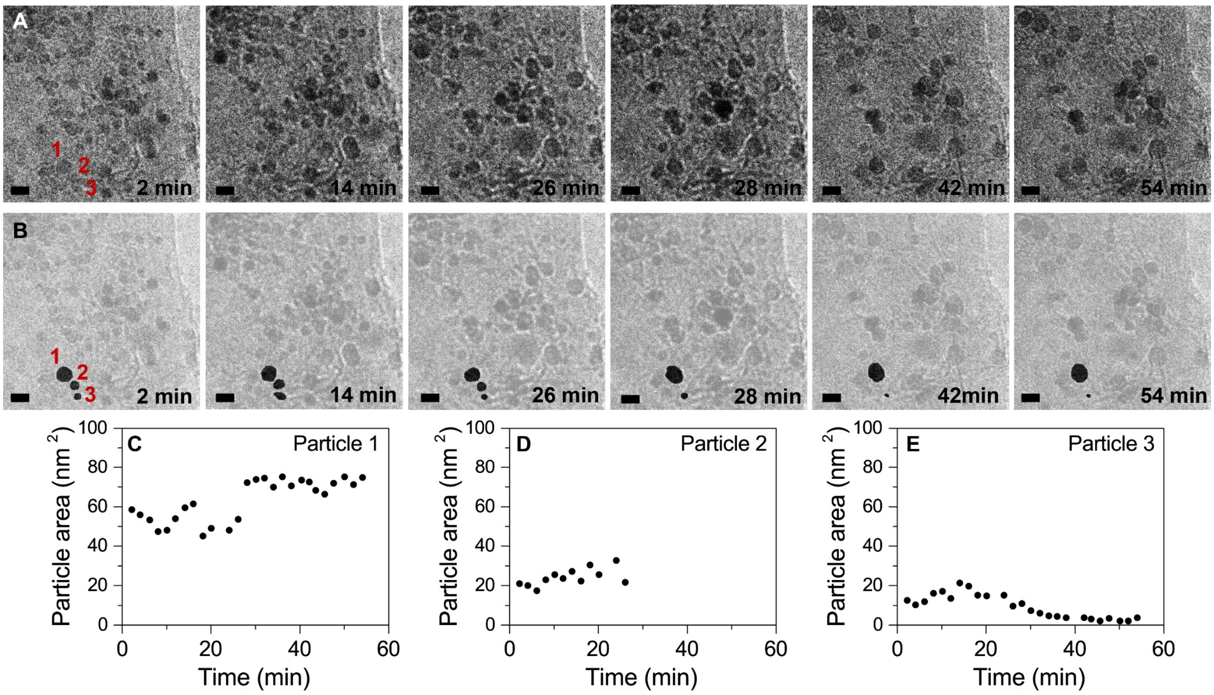

Figure S16. Second example of particle growth via both coalescence and Ostwald ripening. **A)** TEM images with **B)** overlaying masks highlighting the changes of four individual particles over time acquired during *in situ* exposure to 1 bar 4:1 H_2/CO_2 gas with flow of 0.4 sccm at 450 °C with exposure to an electron dose of 8 e^- A^{-2} s^{-1} for ~10 s every 2 min. Scalebar = 10 nm. **C)** Projected area of these particles versus time.

Section 10. Additional figures for experimetal methods

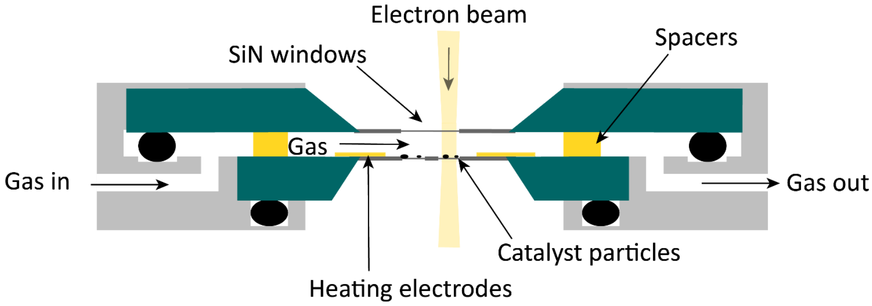

Figure S17. Schematic representation of a gas cell nanoreactor for *in situ* electron microscopy.

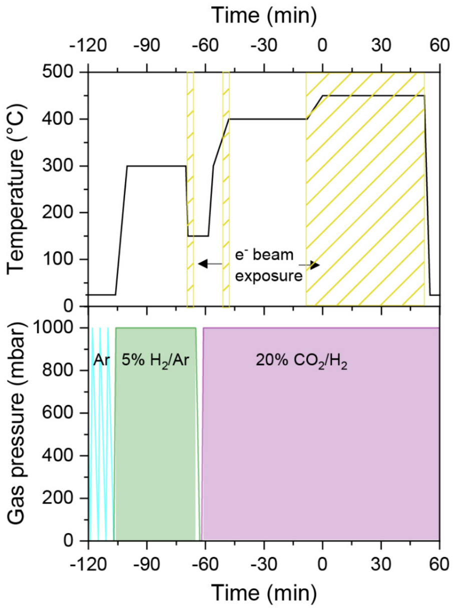

Figure S18. Typical method used for the *in situ* electron microscopy experiments. During the actual experiment (from t = 0 min), the sample was either exposed to the e^- beam for *ca.* 10 seconds every 2 minutes with the beam turned off in between, or the sample was continuously exposed to the e^- beam.

Section 11. Supporting references

(1) Lindner, J.; Ross, U.; Roddatis, V.; Jooss, C. Langmuir Analysis of Electron Beam Induced Plasma in Environmental TEM. *Ultramicroscopy* **2023**, *243* (October 2022), 113629. https://doi.org/10.1016/j.ultramic.2022.113629.

(2) Mølhave, K.; Gudnason, S. B.; Pedersen, A. T.; Clausen, C. H.; Horsewell, A.; Bøggild, P. Electron Irradiation-Induced Destruction of Carbon Nanotubes in Electron Microscopes. *Ultramicroscopy* **2007**, *108* (1), 52–57. https://doi.org/10.1016/j.ultramic.2007.03.001.
